# Tracking Se Assimilation and Speciation through the Rice Plant – Nutrient Competition, Toxicity and Distribution

**DOI:** 10.1371/journal.pone.0152081

**Published:** 2016-04-26

**Authors:** Alexandra K. Nothstein, Elisabeth Eiche, Michael Riemann, Peter Nick, Lenny H. E. Winkel, Jörg Göttlicher, Ralph Steininger, Rita Brendel, Matthias von Brasch, Gabriele Konrad, Thomas Neumann

**Affiliations:** 1 Institute of Applied Geosciences (AGW), Karlsruhe Institute of Technology (KIT), Adenauerring 20b, D-76131 Karlsruhe, Germany; 2 Molecular Cell Biology, Botanical Institute, and Center for Functional Nanostructures (CFN), Karlsruhe, Institute of Technology (KIT), Kaiserstraße 2, D-76131 Karlsruhe, Germany; 3 Eawag, Swiss Federal Institute of Aquatic Science and Technology, CH-8600 Dübendorf, Switzerland; 4 Institute of Biogeochemistry and Pollutant Dynamics, ETH Zürich, Universitätsstraße 16, CH-8092 Zürich, Switzerland; 5 ANKA Synchrotron Radiation Facility, Karlsruhe Institute of Technology (KIT), Hermann-von-Helmholtz-Platz 1, D-76344 Eggenstein-Leopoldshafen, Germany; INRA, FRANCE

## Abstract

Up to 1 billion people are affected by low intakes of the essential nutrient selenium (Se) due to low concentrations in crops. Biofortification of this micronutrient in plants is an attractive way of increasing dietary Se levels. We investigated a promising method of Se biofortification of rice seedlings, as rice is the primary staple for 3 billion people, but naturally contains low Se concentrations. We studied hydroponic Se uptake for 0–2500 ppb Se, potential phyto-toxicological effects of Se and the speciation of Se along the shoots and roots as a function of added Se species, concentrations and other nutrients supplied. We found that rice germinating directly in a Se environment increased plant-Se by factor 2–16, but that nutrient supplementation is required to prevent phyto-toxicity. XANES data showed that selenite uptake mainly resulted in the accumulation of organic Se in roots, but that selenate uptake resulted in accumulation of selenate in the higher part of the shoot, which is an essential requirement for Se to be transported to the grain. The amount of organic Se in the plant was positively correlated with applied Se concentration. Our results indicate that biofortification of seedlings with selenate is a successful method to increase Se levels in rice.

## Introduction

The micronutrient selenium (Se), is essential for all mammals due to its presence in seleno-proteins [1]. A balanced Se-status correlates with reduced incidence of viral infections, auto-immune thyroid diseases, as well as some cancers. However, an excess of Se supplementation has been shown to raise the risk for type-2 diabetes [2]. Characterized by a narrow gap between malnutrition (<55 μg/d [3]) and toxicity (> 400 μg/d [4]), Se has, therefore, been compared to a ‘double-edged sword’ [5].

Considering the fact that an estimated 0.5–1 billion people worldwide suffer from low Se intake [6], Se-biofortification of cereals is a topic of major impact [7,8]. As primary staple crop for more than 3 billion people, rice is considered to be the ‘cereal crop of the world’s poor’ [9]. Detailed molecular mapping and extensive collections of mutants and transgenic lines have established rice as central model for functional genomics in cereals [10]. However, a worldwide survey of Se content in rice grain indicates that for a daily consumption of 300 g of rice per day, 130 ng/g Se are required to account for 70% of the RDI (55 μg/d), yet the world mean concentration in rice grain is around 50 ng/g Se and, therefore, too low to meet recommended human requirements [4]. As rice is such an important food crop, biofortification with Se is a promising means to increase dietary Se levels.

Previous research on Se-uptake into rice has focused on Se uptake from Se-supplemented soils [4,11]. In addition, biomolecular studies on Se transport between plant tissues, or single-ion competition studies are available for rice [12,13]. Unfortunately, how rice takes up the different forms of Se [1,8], and which mechanisms are responsible for Se phyto-toxicity, remains poorly understood [14]. To achieve effective, yet safe, Se-biofortification, Se-uptake into the plant, phyto-toxicological effects of Se, transport and partitioning of Se species within the rice plant have to be investigated [1,12,14]. Furthermore, it is crucial to know how different concentrations and forms of Se in the soil solution affect speciation of Se in the different parts of the plant and in the rice grain [4], as these factors largely control the efficiency of transfer into the bloodstream upon ingestion [5].

XANES (X-Ray Absorption Near Edge Spectra) analysis is a useful technique to study in-situ speciation of Se in plant tissues [15]. So far, there have been only a few XANES studies on Se speciation in rice tissue [8,13,15,16], addressing different research questions. One of these studies investigated Se transport into rice grains [13] and reported that SeMet and SeMeSeCys are the two Se species that can enter the rice grain, while selenite cannot. However, in that study, Se (126.6 μM) was applied via excision below the panicle, which does not represent typical plant Se uptake. Another study [7] analysed Se speciation in rice seedlings grown in hydroponic cultures, employing a combined fluorescence mapping & XANES analyses in hydrated plant tissue and showed that selenate and selenite were converted to C-Se-C compounds in the roots, while free selenate was transported to the shoots. However, that study did not investigate Se speciation along the length of the plant and was only conducted for a single concentration of Se concentration added as selenite or selenate (1 μM Se).

Crop fertilization is often achieved by adding inorganic Se to soils combined with fertilizer. Unfortunately, this is potentially a very wasteful method of Se-biofortification, as 80–95% of Se added as selenate may be washed out due to irrigation or rainfall [17] and selenite is less bioavailable due to adsorption onto ferric soil minerals [17] or accumulation in plant tissue which is not part of human diet, like rice roots [13]. Moreover, rice typically grows in paddy fields, where reducing conditions prevail and thus reduced selenium species such as elemental Se or selenides are likely predominant, which are less bioavailable to plants [10].

Therefore, we investigated Se accumulation upon germination in a Se-rich environment, which was proposed as an idea to achieve biofortification before planting [18]. Liu and Gu [18] showed that Se uptake upon germination was successful. However, long-term effects (> 48h) of Se uptake on potential toxicity or speciation in the rice plants were not investigated. Therefore, in the present study, we investigated both Se uptake and toxicity in rice seedlings after 16 day exposure to different Se concentrations (as selenite and selenate) prior to germination in the presence or absence of nutrient solution. XANES analysis and fluorescence mapping was used to investigate Se concentration and speciation over the whole length of the plant, specifically examining speciation changes between root and shoot. This study provides new insights into the mechanisms of Se uptake and speciation in rice as well as optimum Se speciation and concentration ranges for safe and effective biofortification of Se in rice prior to germination.

## Materials and Methods

### Set-up 1: Germination of rice on Se-spiked nutrient-free phytoagar

**Fig 1:**
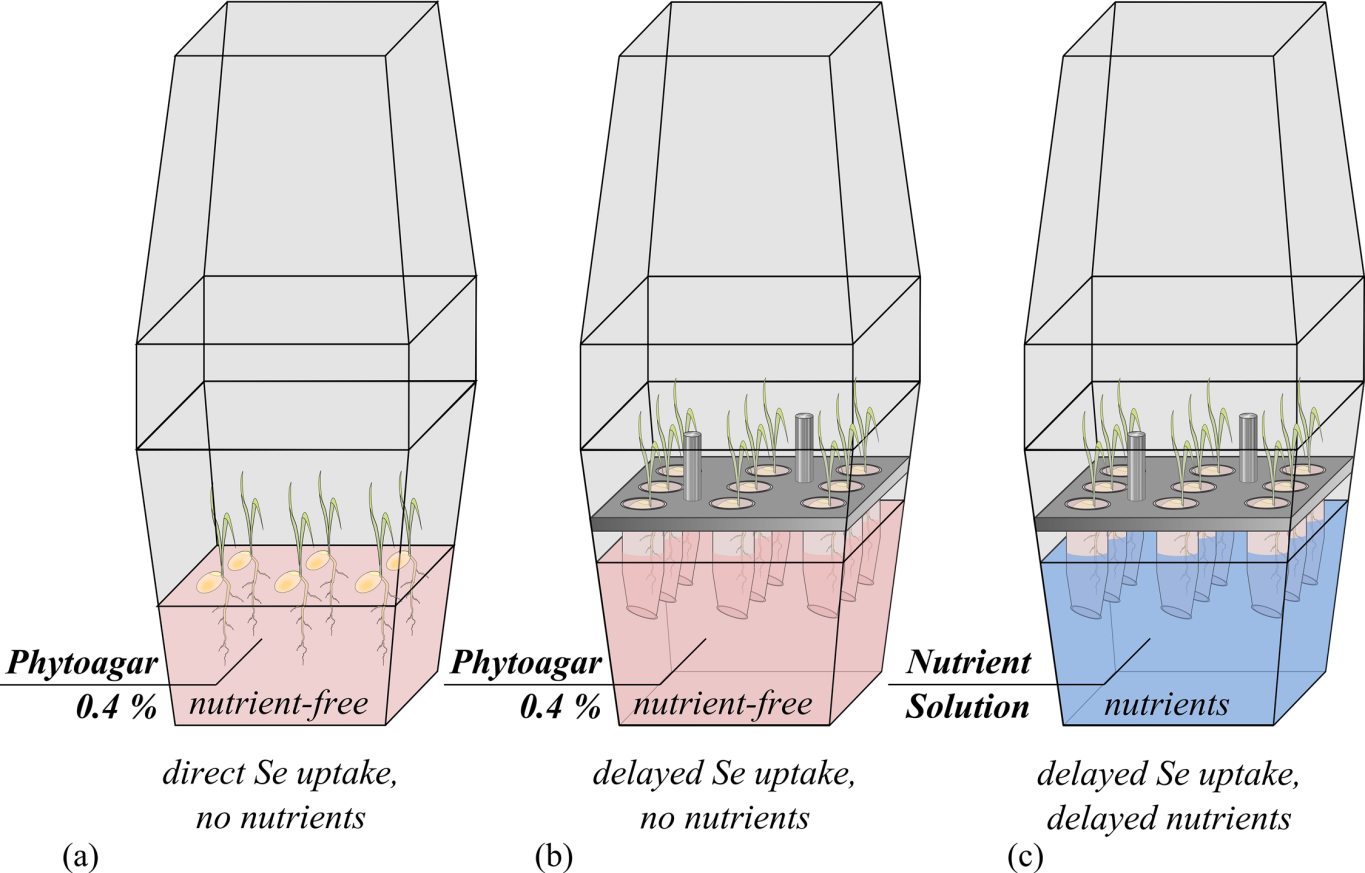
Experimental set-ups for nutrient-free, direct Se exposure (a), nutrient-free, delayed Se exposure (b) and Se-nutrient solution, delayed Se exposure experiments (c).

Plant-box experiments were carried out to investigate Se uptake into rice during the stage of germination in a nutrient-free environment (experimental set-up 1; Fig 1A). Six rice (*Oryza sativa L*. *ssp*. *japonica cv*. *Nihonmasari*) caryopses per box were surface-sterilized with ethanol (80%) and NaOCl (5%) [13], and planted into 100 mL of 0.4% phytoagar (Duchefa Direct) supplemented with Se (Na_2_SeO_4_*10 H_2_O, VWR BDH Prolabo 302113L, or Na_2_SeO_3_, AlfaAesar 012585) in concentrations of 0, 5, 10, 25, 50, 100, 250, 500, 1000 and 2500 μg/L Se in closed Magenta-boxes (Sigma Aldrich, Art. No. V8380, V8505 & C0667). Boxes were kept closed for the duration of the experiment (16 days) in a climate chamber at 70% humidity, with a day-night cycle (daylight: 8 a.m.–4 p.m.) and a transition period of 1 hour for dawn and dusk, respectively, and corresponding temperatures of 28°C (day) and 22°C (night). Three independent biological replicas were conducted of this experiment.

### Set-up 2: treatment of rice with Se-spiked nutrient solution after pre-germination in Se-free conditions

To study the effect of nutrient supplementation on Se-uptake after pre-germination in the absence of Se, (experimental set-up 2; Fig 1C), 9 sterilized plants per box germinated in 0.7% phytoagar (in a reaction tube cut open at the bottom) containing no Se or nutrients, until (after 5 days) the roots reached 170 mL of an optimized nutrient solution [19]: 2500 μM Ca(NO_3_)_2_*4H_2_O, 375 μM K_2_SO_4_, 325 μM MgSO_4_*6H_2_O, 400 μM KH_2_PO_4_, 8 μM H_3_BO_3_, 0.4 μM CuSO_4_, 0.75 μM ZnSO_4_*H_2_O, 1.2 μM MnSO_4_*H_2_O, 50 μM CaCl_2_, 0.075 μM Na_2_MoO_4_*2H_2_O, 75 μM C_6_H_5_O_7_Fe (in double-distilled water) supplemented with 0, 5, 10, 25, 50, 100, 250, 500, 1000 and 2500 μg/L Se. To make the Se-uptake time frame comparable to set-up 1, all plants were harvested after 19 days to compensate for the time required for roots to reach the Se-nutrient solution. Three independent biological replicas were conducted of this experiment.

### Set-up 3: treatment of rice with Se-spiked phytoagar after pre-germination in Se-free conditions

In a variation of set-up 2 (Fig 1B), seedlings were pre-germinated Se- and nutrient-free, but then Se was added to nutrient-free 0.4% phytoagar. This experiment was conducted once for selenate and selenite, respectively.

### Sampling and sample preparation for Se analysis

All plants were harvested, rinsed externally with Millipore water and separated above the caryopses into root and shoot, which were weighed separately to determine fresh weight and freeze-dried at 0.05 mbar and -20°C for 24 h to determine plant dry weight. For plant digestion [20], each bulk sample (roots or shoots per plant-box: 0.01–0.1 g) was digested with 1 mL of double-distilled water, 3 mL of concentrated HNO_3_ (suprapure) and 1 mL of 30% H_2_O_2_ (p.a.) Teflon vessels. Each batch of 10 digestion samples included one blank and one plant standard (0.1 g NBS SRM 1567a Wheat Flour) to verify digestion quality.

### Se analysis with HG-FIAS

Total Se-content of the roots and shoots of plants harvested from all three set-ups was analysed with HG-FIAS (Hydride Generation Flow Injection Atomic Absorption Spectroscopy; Perkin Elmer AAnalyst200, FIMS-400 Hydride Generation System); Total Se in samples was completely reduced to selenite in 6 M Hg-free HCl (Merck, 37%, 1.13386.2500) in a water bath pre-heated to 75°C for 15 min and then diluted to 1 M HCl with double-distilled water and measured with HG-FIAS. For calibration, 10 mL of Se standard solution (1000 μg/L Se, Roth Rotistar ICP) were reduced to selenite in 6 M HCl in the same way as the samples. From this solution, calibration concentrations of 0.5, 0.75, 1, 2, 4, 5, and 6 μg/L Se were prepared with 1 M Hg-free HCl. Reduction quality and drift correction was analyzed using a multi-element drinking water standard (PromoChem Trace Metals QCP 050–1 and QCP 050–2 combined, with 252 μg/L Se).

Quality measures for all three experiments show that harvested plant yield was 86% (± 6), 67% (± 7) and 60% (± 17), respectively, of transferred seedlings. Of the plant standard used to verify digestion quality, 85% (± 14), 87% (± 10) and 98% (± 6), were retrieved, respectively. Drinking water standard retrieval verifying HG-FIAS measurement was 102% (± 7), 104% (± 5) and 118% (± 7), respectively.

### XANES analysis

To determine Se speciation in shoots and roots, using X-ray absorption near edge structure (XANES), set-up 2 was repeated for Se concentrations of 500, 2000 and 10,000 μg/L Se as selenite and selenate. Prior to analyses, roots and shoots were air-dried in a desiccator for 4 weeks to avoid Se-loss and speciation change and to preserve plant structures during the drying process. Roots and shoots fixed on Kapton tape were measured at the SUL-X beamline (Synchrotron facility ANKA, Karlsruhe) from -75 eV to +200 eV around the absorption edge of 12.658 keV in fluorescence mode under vacuum. Preliminary experiments had verified that there were no significant differences between N_2_-cryo-cell or vacuum measurements.

Per plant cross section, 5–8 scans were measured with short acquisition times (ca. 7 min), which were merged for better accuracy. To minimize beam-induced redox reactions, a maximum of 2 scans was performed on the exact same spot (beam spot on sample: 30 x 50 μm). References for combination fitting between -30 eV and +30 eV around the absorption edge (Athena, Demeter Software 0.9.13) were measured in transmission mode as powders on Kapton tape of seleno-methionine (Sigma Aldrich S3132-100 MG), Na_2_SeO_3_ (AlfaAesar 012585) and Na_2_SeO_4_ • 10H_2_O (VWR BDH Prolabo 302113 L).

For energy calibration, all samples and references were measured against the Se(0) standard prepared as a pressed pellet with 8 mg of elemental Se (Merck 1.07714.0050) in 100 mg of cellulose powder (Sigma Aldrich C8002-1KG). The SUL-X beamline was equipped with a 7-element fluorescence detector (2 eV; Resolution: <310 eV at 5.9 keV, 100.000 cps), three ionisation chambers for absorption measurement (Oxford Instruments, IC-Plus type; active length 5 cm, Kapton windows 6 μm thick), a CCD detector (Photonic science; 80 x 120 mm 2, Fiber optic 3.46:1, 2048 x 2048, 16 bit dynamic, readout time 3.3 s 21 s) and an optical microscope (TSO Spezialoptik, resolution 2 μm).

### High resolution μ-XRF mapping

To determine the distribution of Se within the plant, X-ray fluorescence (XRF) was mapped in the roots and shoots at an excitation energy of 22 keV at the FLUO beamline at ANKA, Karlsruhe, equipped with a double multilayer monochromator (W-Si multilayers in 2.7 nm period), CRL: 2x10 9 ph/s at 17 keV (5 μm x 2 μm), 1 ionisation chamber, Si(Li)-energy dispersive detector (Oxford Instruments), HPGe-High Purity Germanium detector (Princeton Gamma-Tech (PGT)), and a SiMCD-Vortex Silicon Multicathode Detector. Two undiluted, pressed pellets of bulk plant material (Brassica juncea) from Punjab, India [21] with known Se concentrations (measured with ICP-MS after acid digestion) were used to calibrate Se concentration in the mappings (root: 186 mg/kg Se DW; leaf: 931 mg/kg Se DW). Plant samples were placed between Kapton tapes to ensure a plane surface. Se fluorescence meshs (at 11.2222 keV for kα1) across plant sections (measured across the point from -1 mm to +1 mm in 50 steps for 1 s) were measured at an excitation energy of 22 keV. Beam spot size on the sample was 16 x 11 μm (polycapillary focus), and acquisition time was 3–4 sec. Data were analysed using the PyMCA (version 3.9.5) software and mappings were created with Surferplot 6 (Golden Software).

## Results and Discussion

### Se uptake—influence of nutrient supply and timing of Se application

**Fig 2:**
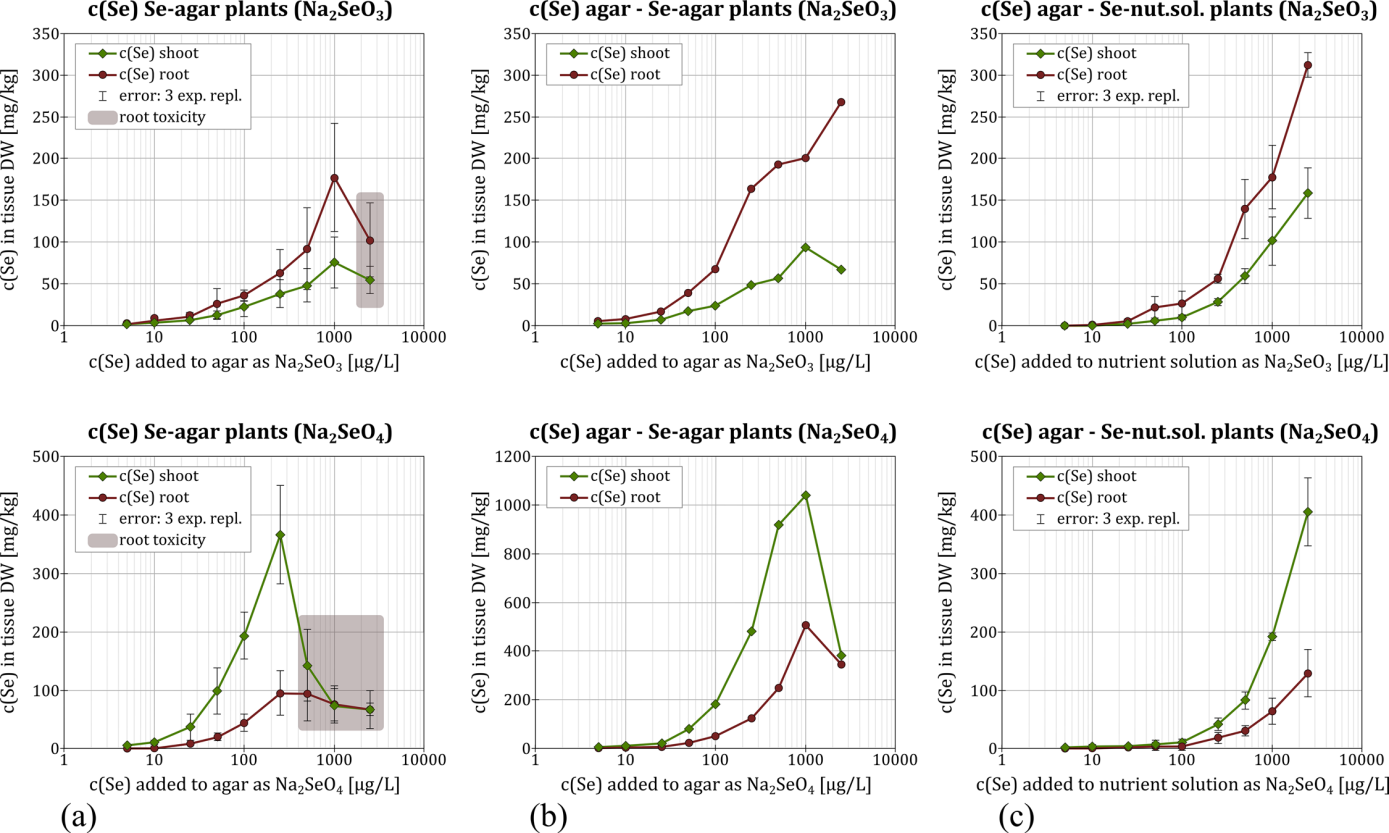
Uptake of applied Se into rice plants expressed as Se concentration in mg per kg of dry plant matter for all three experimental set-ups: nutrient-free, direct Se exposure (a), Se-nutrient solution, delayed Se exposure (b) and nutrient-free, delayed Se exposure experiments (c).

One-way ANOVA testing (n = 3, k = 9) revealed that the addition of Se significantly influenced Se content in the rice plants with P < 0.001 in all experiments (S1–S8 Tables).When rice plants were grown in the nutrient-free environment but exposed to Se directly upon germination (Fig 1A), greater Se content in the agar resulted in greater Se content in plant tissue. This was valid for selenate additions up to 250 μg/L Se, resulting in 367 mg/kg DW Se in shoots and 96 mg/kg DW Se in roots (Fig 2A) and for selenite additions up to 1000 μg/L Se, resulting in 75 mg/kg DW Se in shoots and 177 mg/kg DW Se in roots (Fig 2A). When Se concentration exceeded these values, however, Se content decreased again for both selenate (67 mg/kg DW Se in shoots and roots) and selenite (55 mg/kg DW Se in shoots and 102 mg/kg DW Se in roots). This was accompanied by symptoms of toxicity, such as diminished shoot height (55 and 44% of untreated growth), stunted root growth (root growth < 3 mm vs. 5–8 cm), lack of secondary roots and brown discoloration (S2 Fig).

When rice plants were pre-germinated in the nutrient-free environment, but exposed to Se 5–7 days after germination (Fig 1B) Se content in tissue increased with higher concentrations of Se in the medium (Fig 2C). For selenate, 1000 μg/L Se resulted in 1040 mg/kg DW Se in shoots and 505 mg/kg DW Se in roots; for selenite this concentration resulted in in 170 mg/kg DW Se in the shoots, and 93 mg/kg DW Se in the roots. This treatment thus resulted in greater Se accumulation in the plant tissue for all Se additions (by a factor of about 1.2–5.2) compared to the first experiment, where plants had germinated in a Se environment. Moreover, plants exhibited no toxicity symptoms, even when adding Se concentrations greater than 1000 μg/L Se. At concentrations higher than that, however, tissue content of Se was lower—especially for treatment with selenate (382 mg/kg DW Se in shoots and 345 mg/kg DW Se in roots), but also for root Se-content in response to treatment with selenite (67 mg/kg DW Se). In contrast, shoot Se-content increased to 269 mg/kg DW Se for the addition of 2500 μg/L Se. μXRF mapping showed that addition of 10,000 μg/L Se resulted in highest Se concentrations in the vascular bundles (S7 Fig) with Se content being 6 times higher compared to the surrounding parenchymatic tissue.

The pattern of uptake differed when rice plants were first pre-germinated in the absence of Se and then exposed to Se after a delay of 5–7 days after germination while being supplied with nutrients (Fig 1C). Here, Se-content increased steadily with greater concentrations of Se for both selenate (405 mg/kg DW Se in shoots and 128 mg/kg DW Se in roots) and selenite (159 mg/kg DW Se in shoots and 312 mg/kg DW Se in roots).

### Nutrient supply controlled Se-uptake

Our data show increased Se-uptake in the absence of nutrients (Fig 2A and 2C) compared to experiments with nutrient solutions (Fig 2C) up to 250 μg/L Se, irrespective of the speciation. This antagonism between Se uptake and nutrient supply might be attributed to two phenomena: (1) Lacking competition for free binding sites at transporters involved in Se uptake (sulphate, phosphate or silicon transporters [11,12]) might increase Se-uptake passively. It is known that additional nutrient elements, especially phosphate and sulphate influence Se-uptake by plant roots [1,11], although effects of sulphate are considered more prominent then those of phosphate, because the affinity of selenate to the sulphate transporter is higher than that of selenite to the phosphate transporter [11,22]. (2) Active up-regulation in number or activity of sulphate, phosphate or silicon transporters in response to low abundance of these nutrients in the medium, which as a consequence would also increase Se-uptake [1].

For higher Se concentrations (≥ 500 μg/L Se), the presence of nutrients led to higher uptake of Se compared to nutrient-free treatment. Though a higher dose of Se had to be added than in the nutrient-free experiments to achieve the same plant Se-content, plants in nutrient solution remained healthy. Moreover, they even took up more Se than any of the plants raised in the absence of nutrients treated with Se from germination (Fig 2B). This promotion of Se uptake by nutrients might be linked with the protective effect of S against Se toxicity reported in the literature [23]. Therefore, we conclude that nutrient supply likely increases plant resilience due to anion competition during uptake into the plant, or during subsequent Se transport within the plant, and the incorporation of Se versus S into proteins [11].

### Nutrient-free cultivation only induces phyto-toxicity when plants pre-germinate in Se

**Fig 3:**
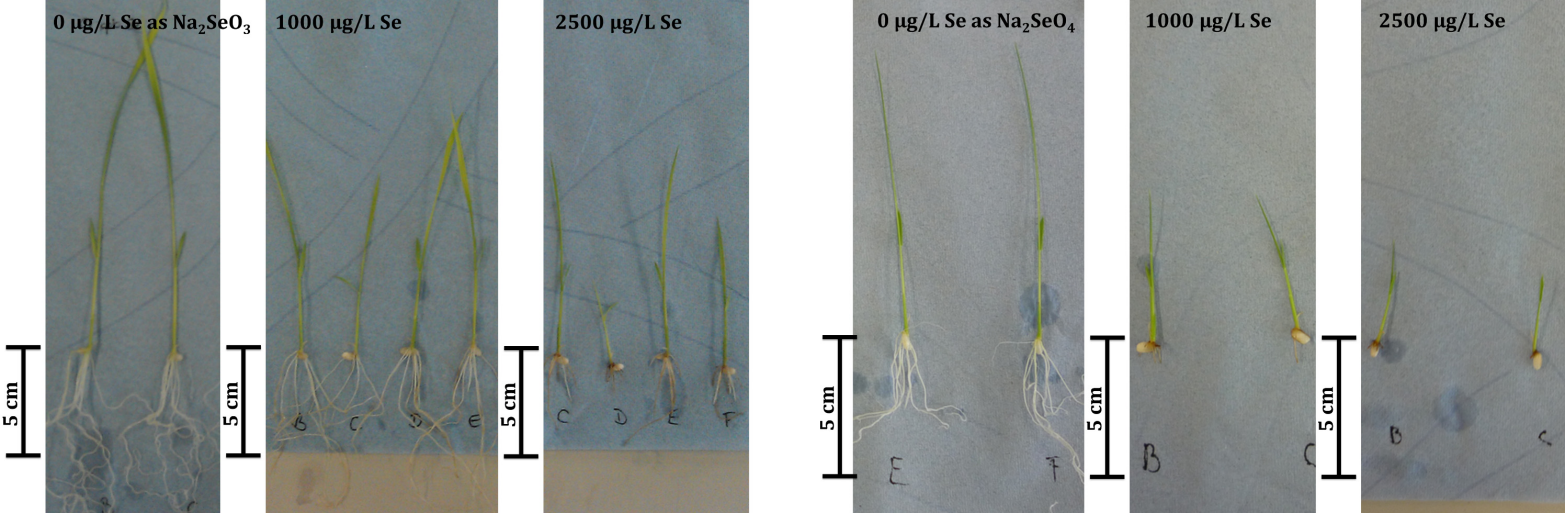
comparison of rice root growth for the additions of 0, 1000 and 2500 μg/L Se as Na_2_SeO_3_ or Na_2_SeO_4_ to agar of the nutrient-free, direct Se exposure experiment.

None of the plants with a 5-day delay of Se exposure after germination (irrespective of the presence or absence of nutrients) showed signs of toxicity. Only rice plants directly exposed to Se during germination in the absence of nutrients exhibited a decrease in tissue Se-content coupled with phyto-toxicity and impaired root development at concentrations of ≥ 250 μg/L Se as selenate or ≥ 1000 μg/L Se as selenite (Figs 2A and 3). We, therefore, concluded that root functionality and, thus, uptake of Se was impaired. Since both effects can be mitigated by addition of nutrients, competition of Se and essential ions for transporter proteins might be the primary cause for the observed Se toxicity. Our study strongly suggests that Se bio-fortification administered to germinating seedlings has to be conducted in the presence of nutrients to avoid phytotoxicity and lowering crop yield already at low Se concentrations. Up until now, effects of Se toxicity and bio-fortification have only been tested on healthy, pre-germinated seedlings [7,11,20]. Only one study [18] directly addressed germination of rice in Se, however, not beyond radicle emergence and therefore our study investigated for the first time subsequent seedling development.

### Se speciation—partitioning and distribution within the plant

**Fig 4:**
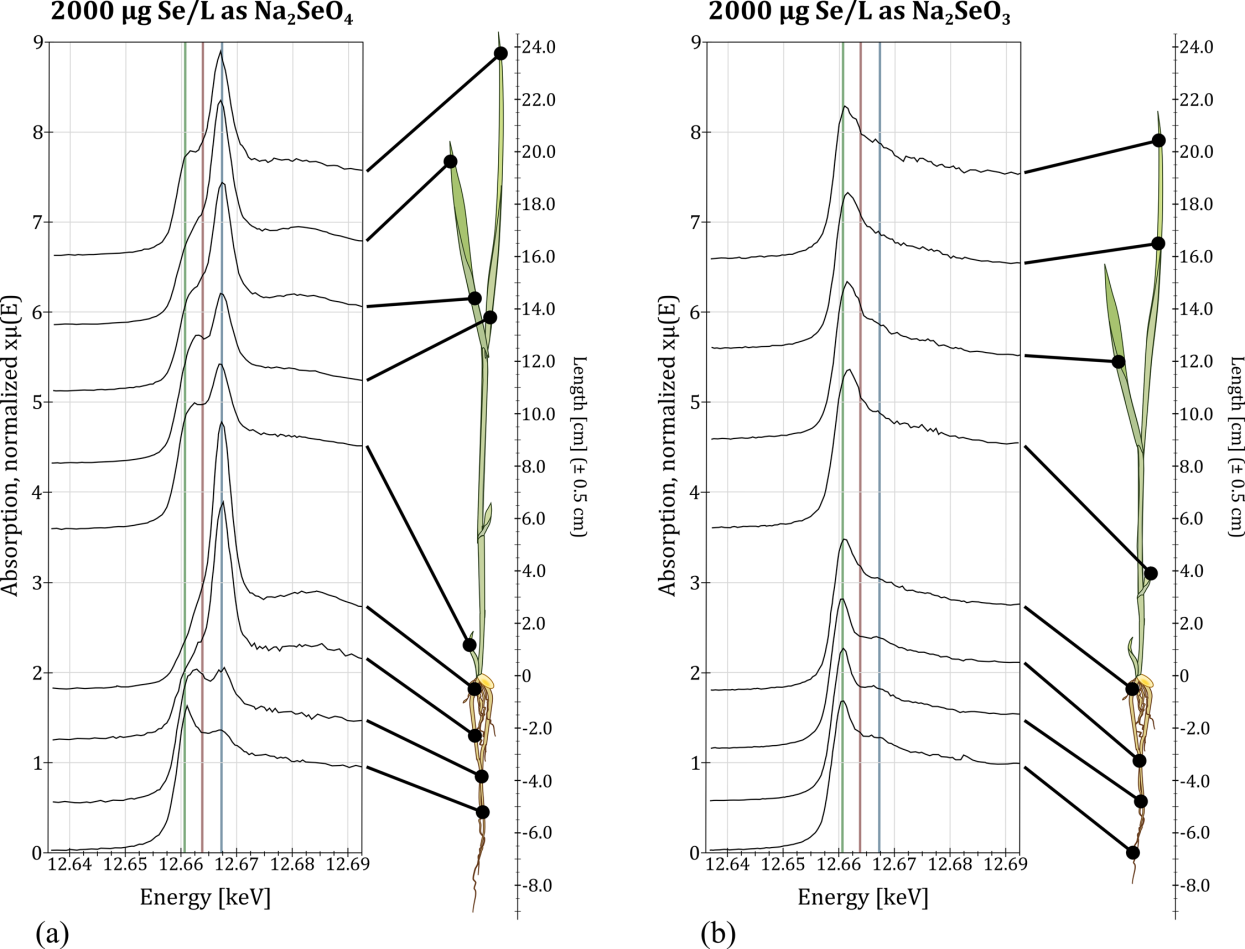
XANES results in shoot and root of a dried rice plant treated with 2000 μg/L Se as Na_2_SeO_4_ (a); or Na_2_SeO_3_ (b) with green, red & blue indicating peak lines for selenomethionine (12.661 keV), selenite (12.664 keV) and selenate (12.667 keV), respectively.

Independent of the mode of cultivation, when adding Se as selenate, Se was preferentially partitioned to the shoots (factor 3.5–5, 72% of total plant-Se on average) rather than the roots (Fig 2). Selenium speciation was inhomogeneous, particularly in the root (Fig 4), with selenate dominant near the caryopses while organic Se was more dominant in lower parts of the root (linear combination fitting confirmed this S12 Table). In the shoots, selenate uptake led to enrichment of selenate toward the leaf ends of higher leaves, compared to organic Se in lower leaves.

When Se was added as selenite, Se accumulated more in the roots (factor 2–4, 70% of total plant-Se on average) than in the shoots (Fig 2, S9 Table). Furthermore, an increase of Se content in the medium corresponded to an increase of organic Se in the plant tissue, irrespective of the added speciation, while proportions of selenate and selenite in the plant tissue decreased accordingly.

This inverse partitioning has also been reported for other cereals, such as perennial ryegrass [22], and wheat [11]. Our findings are, therefore, consistent with the current explanation that selenate is taken up actively by the sulphate transporter and readily transported via the xylem into plant shoots and leaves, while selenite is rapidly transformed into organic Se already in the roots [7,11,22].

In contrast to other results [16], however, our data show speciation proportions in the rice plant to be dependent on Se concentration as well as added species. Not only did higher Se concentrations lead to a greater share of organic Se in the plant tissue, but Se speciation in the medium determined Se speciation distribution within the plant: selenite addition led to uniformly distributed Se (mainly organic Se) throughout the plant, while resulting speciation within the plant was highly inhomogeneous when Se was added as selenate (Fig 4).

While the mechanisms of selenite uptake and inner-plant transport are not fully understood, particularly with respect to the role of phosphate [11] and silicone transporters [1,12], there is consensus on the fact that selenite is rapidly converted into organic Se in the roots [1,21,22], as is also shown by our data (Fig 4). The higher proportions of organic Se in the root compared to the shoot can be explained by Se being stored as organic Se in the roots. This does not necessarily imply incorporation into proteins, since it could also be present as free amino acid, such as MeSeCys [24]. In Arabidopsis, this amino acid has been shown to accumulate in chloroplasts and the SeCys-methylation is thought to reduce toxicity-inducing mis-incorporation of SeCys instead of the S-containing Cys into proteins [3,24–26]. We believe this mechanism explains both the generally high proportion of organic Se within the plant as well as the preferential accumulation of selenite in the root by conversion into methylated or unmethylated forms of SeCys.

### Selenite transport into rice shoots

As for the Se transported into the shoots after selenite treatment, our data indicated the vascular bundles as the most likely path of transport, because Se was found to be enriched in this tissue compared to the surrounding parenchyma (S7 Fig). In what form Se is transported through vascular bundles, however, remains to be elucidated. One possibility would be transport as selenite, which has not yet been transformed into organic Se. This appears plausible, as only 1/4 of the plant-Se was found to travel to the shoot, and this might pose no toxicity threat to the plant. This is supported by the fact that we found no indication of methylated precursors for Se-volatilization [21,25], a common pathway to remove excess Se. Furthermore, we estimate through linear combination fitting that 11–17% of Se were estimated to be present as non-converted selenite within the plant (Table 1). The second possibility might be that Se is transported as an organic Se compound as suggested by Carey et al. [13], who demonstrated quick inner-plant transport of selenomethionine (SeMet) and selenomethylcysteine (SeMeSeCys) in excised rice leaves. These organic compounds moved to the grain exclusively via the phloem, whereas selenate was transported via xylem. Further studies are required to understand how organic Se species are prevented from incorporation into proteins and committed for transport via the phloem.

**Table 1:** Mean linear combination fitting results & R-factor for Se speciation in plant tissue after treatment with 500, 2000 or 10000 μg/L Se as selenate or selenite.

c(Se)	tissue	org. Se	SeO_3_^2-^	SeO_4_^2-^	R-fac.
[μg/L]		[%]	[%]	[%]	[–]
**Se treatment with Na_2_SeO_3_**					
500	shoot	65 ±8	35 ±8	0 ±0	2.26
	root	85 ±9	15 ±9	0 ±0	0.85
2000	shoot	73 ±11	27 ±11	0 ±0	1.84
	root	95 ±4	3 ±5	1 ±1	0.47
10000	shoot	78 ±3	22 ±3	0 ±0	0.81
	root	99 ±2	0 ±1	0 ±0	1.25
**Se treatment with Na_2_SeO_4_**					
500	shoot	38 ±10	14 ±9	48 ±16	1.00
	root	42 ±10	13 ±6	46 ±16	0.88
2000	shoot	45 ±11	18 ±4	38 ±15	0.63
	root	48 ±30	17 ±7	36 ±33	0.68
10000	shoot	54 ±11	13 ±6	32 ±12	0.44
	root	54 ±7	18 ±4	38 ±15	0.63

### Selenate transport into rice shoots

Our data on speciation in the plant after selenate addition indicate that about half of the 1/5th of Se found in the roots is being transported as selenate, while the rest is probably immobilized as organic Se in the root. It has been reported that selenate reduction, which takes place in the chloroplasts, is the rate-limiting step of selenate assimilation, which produces GsH-Se-, SeCys and then SeMet [25]. Since roots are void of chloroplasts (it is considered unlikely that amyloplasts participate in assimilation), it is not surprising that added selenate is mostly transported to the shoots. However, linear combination fitting of our results estimated 13–18% of Se in the shoot to be selenite. Although selenate can be reduced to selenite [3,25], it remains unclear, how this reduction competes with conversion into organic Se, as it has been reported for roots [1]. Alternatively, a different Se species not accounted for by the linear combination fitting, might occur. Selenite in the shoots detected by linear combination fitting of XANES data has also been reported for wheat [21], but was not reported for a similar XANES analysis of rice [7]. Detection of selenite in the shoots might indicate selenite transport to the upper parts of the plant, but this would not agree with the published record [10,16,25,26]. We believe it is more likely that a vital Se reference is missing in the linear fitting, since selenite is not considered part of the selenate assimilation process [25]. As argued previously in the literature [7,16,27], XANES spectra of some organic Se species are difficult to distinguish and resulting linear combination fittings easily show a 10%—error. Several Se compounds that include S could are plausible candidates, since their XANES spectra show peaks at multiple energies between organic species and selenite [28]. Determination of these spatial-speciation differences will require improved spectral resolution.

In conclusion, our results show that Se uptake during the germination stage is a very effective method of rice biofortification with Se. To avoid phyto-toxicity, nutrients must be added as well. To achieve the highest Se content in the rice grain, Se should be applied as selenate rather than selenite. This study has provided insight into mechanisms of rice biofortification with Se and how to more effectively use the available Se resources during this process. Future studies need to address the transport of Se within the plant—particularly regarding transfer pathways and the spatial distribution of organo-seleno compounds. This will require improved spatial and molecular resolution of analytical methods to address the spatial differentiation of Se speciation *in vivo*.

## Supporting Information

S1 Fig Plant growth expressed as percentage of Se-free blank plants, for shoot height and height of the 2^nd^ leaf for all three experimental set-ups: nutrient-free, direct Se exposure (a), Se-nutrient solution, delayed Se exposure (b) and nutrient-free, delayed Se exposure experiments (c).
(PDF)

S2 Fig Comparison of rice root growth for the additions of 0, 1000 and 2500 μg/L Se as Na_2_SeO_3_ or Na_2_SeO_4_ to agar of the nutrient-free, direct Se exposure experiment. (PDF)

S3 Fig XANES of the shoot and root of a dried rice plant treated with 500 μg/L Se as Na_2_SeO_3_ (left) or Na_2_SeO_4_ (right); green, red & blue indicating peak lines for selenomethionine (12.661 keV), selenite (12.664 keV) and selenate (12.667 keV), respectively. (PDF)

S4 Fig XANES of the shoot and root of a dried rice plant treated with 2000 μg/L Se as Na_2_SeO_3_ (left) or Na_2_SeO_4_ (right); green, red & blue indicating peak lines for selenomethionine (12.661 keV), selenite (12.664 keV) and selenate (12.667 keV), respectively. (PDF)

S5 Fig XANES of the shoot and root of a dried rice plant treated with 10,000 μg/L Se as Na_2_SeO_3_ (left) or Na_2_SeO_4_ (right); green, red & blue indicating peak lines for selenomethionine (12.661 keV), selenite (12.664 keV) and selenate (12.667 keV), respectively. (PDF)

S6 Fig Selenium fluorescence mappings of shoot and root tissue of a dried rice plant treated with 10,000 μg/L Se as Na_2_SeO_4_; photos taken with a binocular. (PDF)

S7 Fig Selenium fluorescence mappings of shoot and root tissue of a dried rice plant treated with 2000 μg/L Se (top) and 10,000 μg/L Se (bottom) as Na_2_SeO_3_; photos taken with a binocular. (PDF)

S8 Fig Results for plant Se content for each of the three experimental runs of agar experiments. (PDF)

S9 Fig Results for plant Se content for each of the three experimental runs of nutrient solution experiments. (PDF)

S10 Fig Results for shoot height and length of the 2^nd^ leaf for plants from the agar experiments. (PDF)

S11 Fig Results for shoot height and length of the 2^nd^ leaf for plants from the nutrient solution experiments. (PDF)

S12 Fig Calculated element uptake into plants from nutrient solution plotted against added Se concentration. (PDF)

S13 Fig Photos of harvested plants treated with Na_2_SeO_3_ in phytoagar & direct Se. (PDF)

S14 Fig Photos of harvested plants treated with Na_2_SeO_4_ in phytoagar & direct Se. (PDF)

S15 Fig Photos of harvested plants treated with Na_2_SeO_3_ in nutrients & delayed Se. (PDF)

S16 Fig Photos of harvested plants treated with Na_2_SeO_4_ in nutrients & delayed Se. (PDF)

S17 Fig Photos of harvested plants treated with Na_2_SeO_3_ in phytoagar & delayed Se. (PDF)

S18 Fig Photos of harvested plants treated with Na_2_SeO_4_ in phytoagar & delayed Se. (PDF)

S19 Fig all XANES spectra of each region of interest (ROI) on a rice plant treated with 500 μg/L Se as Na_2_SeO_3_ in nutrient solution. (PDF)

S20 Fig all XANES spectra of each region of interest (ROI) on a rice plant treated with 500 μg/L Se as Na_2_SeO_4_ in nutrient solution. (PDF)

S21 Fig all XANES spectra of each region of interest (ROI) on a rice plant treated with 2000 μg/L Se as Na_2_SeO_3_ in nutrient solution. (PDF)

S22 Fig all XANES spectra of each region of interest (ROI) on a rice plant treated with 2000 μg/L Se as Na_2_SeO_4_ in nutrient solution. (PDF)

S23 Fig all XANES spectra of each region of interest (ROI) on a rice plant treated with 10,000 μg/L Se as Na_2_SeO_3_ in nutrient solution. (PDF)

S24 Fig all XANES spectra of each region of interest (ROI) on a rice plant treated with 10,000 μg/L Se as Na_2_SeO_4_ in nutrient solution. (PDF)

S1 Table One-way ANOVA results for shoot-Se in agar plants when added as selenite. (PDF)

S2 Table One-way ANOVA results for root-Se in agar plants when added as selenite. (PDF)

S3 Table One-way ANOVA results for shoot-Se in agar plants when added as selenite. (PDF)

S4 Table One-way ANOVA results for root-Se in agar plants when added as selenite. (PDF)

S5 Table One-way ANOVA results for shoot-Se in nut.sol. plants when added as selenite. (PDF)

S6 Table One-way ANOVA results for root-Se in nut.sol. plants when added as selenite. (PDF)

S7 Table One-way ANOVA results for shoot-Se in nut.sol. plants when added as selenite. (PDF)

S8 Table One-way ANOVA results for root-Se in nut.sol. plants when added as selenite. (PDF)

S9 Table Se distribution for uptake of selenate and selenite into root and shoot in all three experimental set-ups (dshoot [%] = c(Se)shoot [mg/kg]/(c(Se)shoot [mg/kg] + c(Se)root [mg/kg])*100). (PDF)

S10 Table Wet weight accumulation factors AF of selenate into plant tissue in all three experimental set-ups (AF [–] = c(Se)medium [mg/L] / c(Se)plant [mg/kg]). (PDF)

S11 Table Wet weight accumulation factors AF of selenite into plant tissue in all three experimental set-ups (AF [–] = c(Se)medium [mg/L] / c(Se)plant [mg/kg]). (PDF)

S12 Table Linear Combination Fitting Results using references for selenomethionine (org. Se), selenite and selenite. (PDF)
