# Supplementary material for: Tracking Se Assimilation and Speciation through the Rice Plant – Nutrient Competition, Toxicity and Distribution
Source: PLoS One. 2016 Apr 26;11(4):e0152081. doi: 10.1371/journal.pone.0152081 (PMC4846085; doi:10.1371/journal.pone.0152081)
Supplement: S1 Fig — Plant growth expressed as percentage of Se-free blank plants, for shoot height and height of the 2nd leaf for all three experimental set-ups: nutrient-free, direct Se exposure (a), Se-nutrient solution, delayed Se exposure (b) and nutrient-free, delayed Se exposure experiments (c). (PDF) [file pone.0152081.s001.pdf]

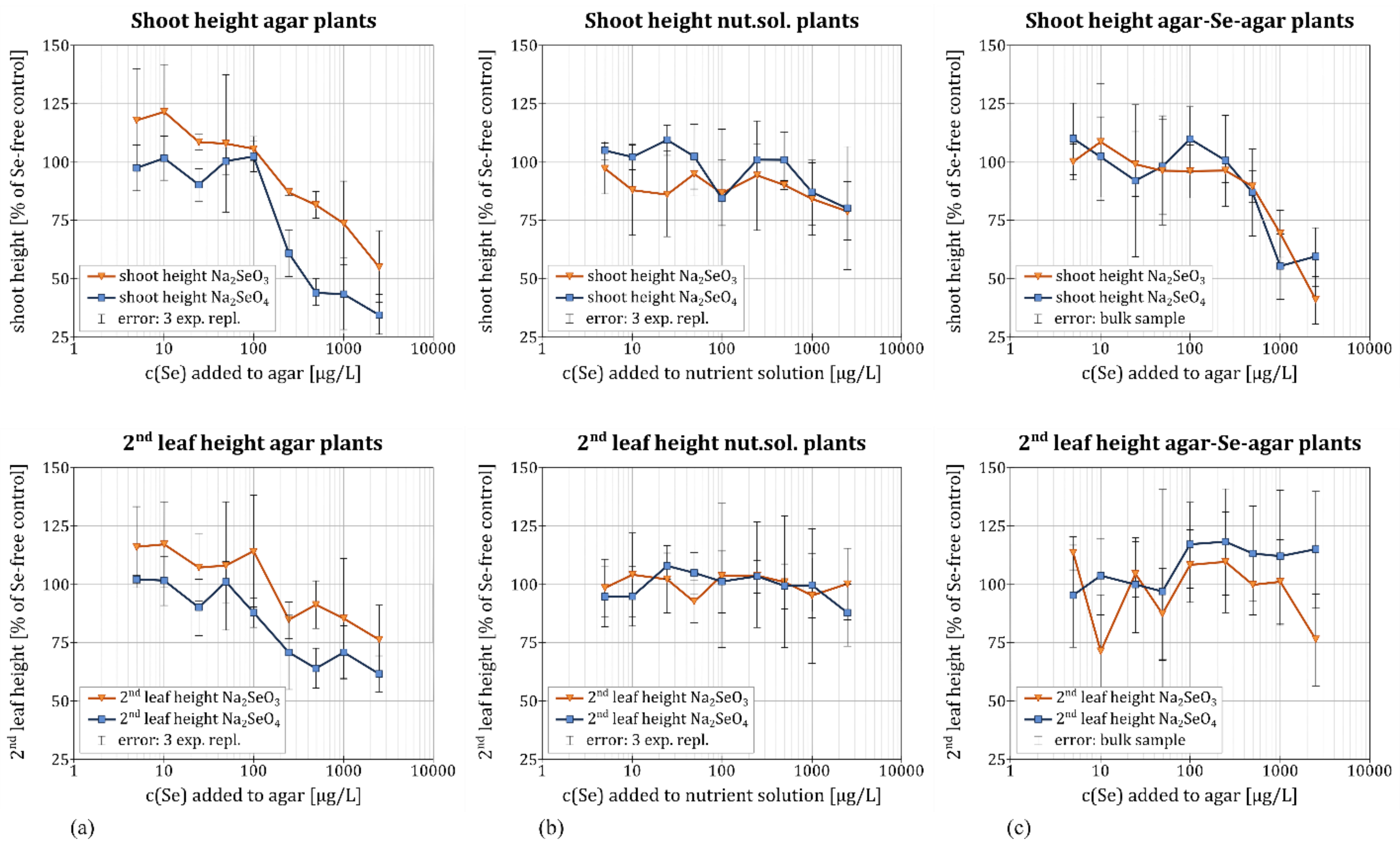

S1 Fig: Plant growth expressed as percentage of Se-free blank plants, for shoot height and height of the 2nd leaf for all three experimental set-ups: nutrient-free, direct Se exposure (a), Se-nutrient solution, delayed Se exposure (b) and nutrient-free, delayed Se exposure experiments (c).
