# Supplementary material for: Tracking Se Assimilation and Speciation through the Rice Plant – Nutrient Competition, Toxicity and Distribution
Source: PLoS One. 2016 Apr 26;11(4):e0152081. doi: 10.1371/journal.pone.0152081 (PMC4846085; doi:10.1371/journal.pone.0152081)
Supplement: S2 Fig — (PDF) [file pone.0152081.s002.pdf]

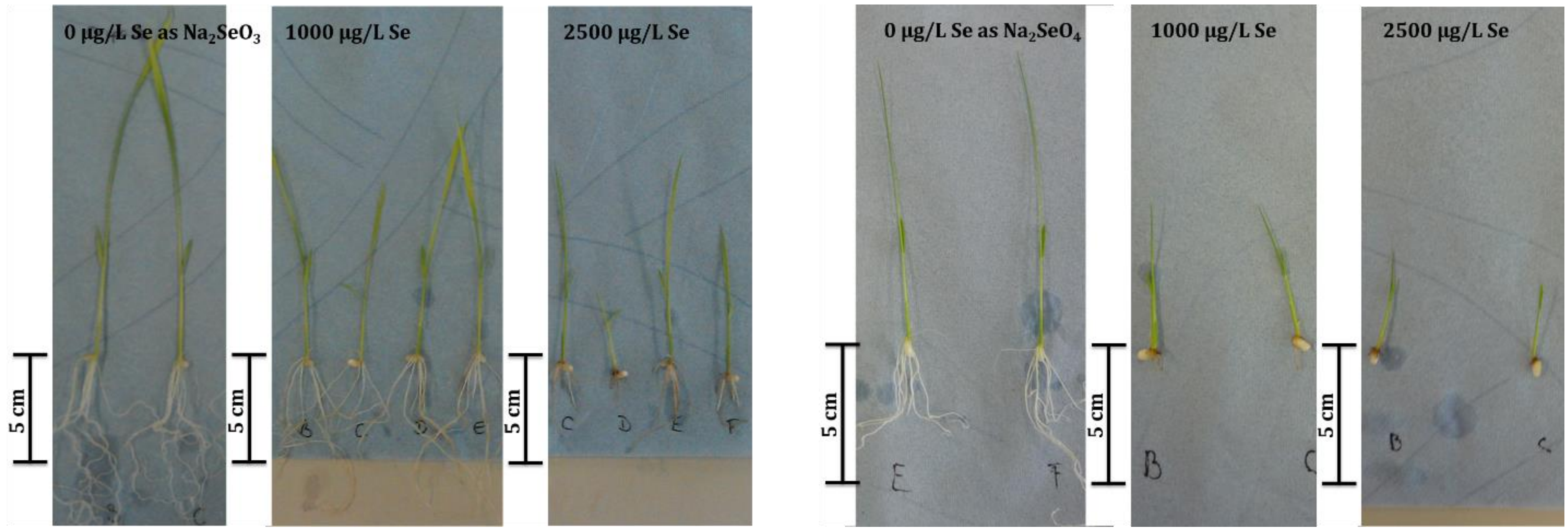

S1 Fig: comparison of rice root growth for the additions of 0, 1000 and 2500 µg/L Se as Na_2SeO_3 or Na_2SeO_4 to agar of the nutrient-free, direct Se exposure experiment
