# Supplementary material for: Tracking Se Assimilation and Speciation through the Rice Plant – Nutrient Competition, Toxicity and Distribution
Source: PLoS One. 2016 Apr 26;11(4):e0152081. doi: 10.1371/journal.pone.0152081 (PMC4846085; doi:10.1371/journal.pone.0152081)
Supplement: S4 Fig — (PDF) [file pone.0152081.s004.pdf]

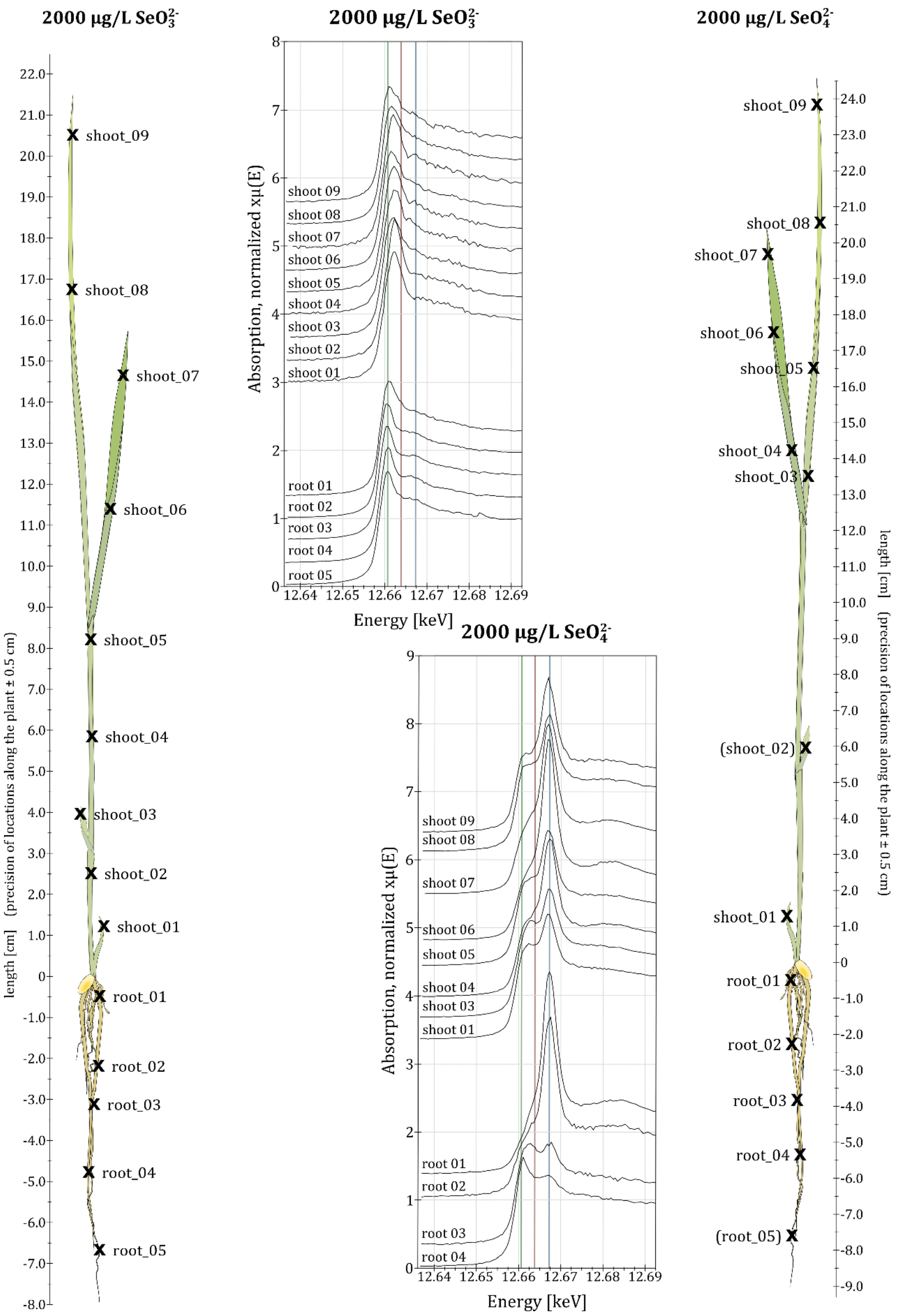

S1 Fig: XANES of the shoot and root of a dried rice plant treated with 2000 µg/L Se as Na_2SeO_3 (left) or Na_2SeO_4 (right); green, red & blue indicating peak lines for selenomethionine (12.661 keV), selenite (12.664 keV) and selenate (12.667 keV), respectively
