# Supplementary material for: Tracking Se Assimilation and Speciation through the Rice Plant – Nutrient Competition, Toxicity and Distribution
Source: PLoS One. 2016 Apr 26;11(4):e0152081. doi: 10.1371/journal.pone.0152081 (PMC4846085; doi:10.1371/journal.pone.0152081)
Supplement: S6 Fig — (PDF) [file pone.0152081.s006.pdf]

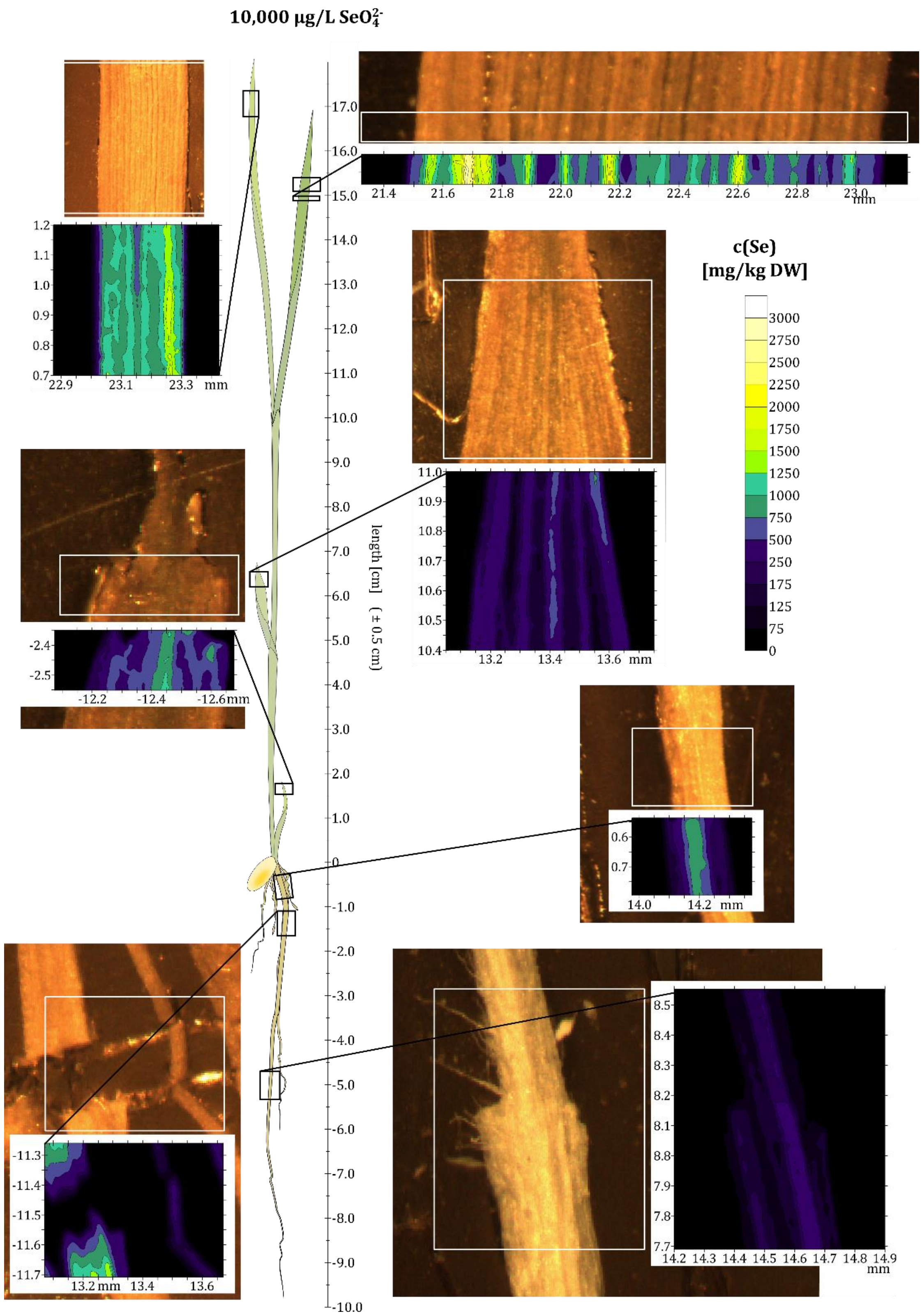

S1 Fig: Selenium fluorescence mappings of shoot and root tissue of a dried rice plant treated with 10,000 µg/L Se as Na_2Se_4; photos taken with a binocular
