# Supplementary material for: Tracking Se Assimilation and Speciation through the Rice Plant – Nutrient Competition, Toxicity and Distribution
Source: PLoS One. 2016 Apr 26;11(4):e0152081. doi: 10.1371/journal.pone.0152081 (PMC4846085; doi:10.1371/journal.pone.0152081)
Supplement: S7 Fig — (PDF) [file pone.0152081.s007.pdf]

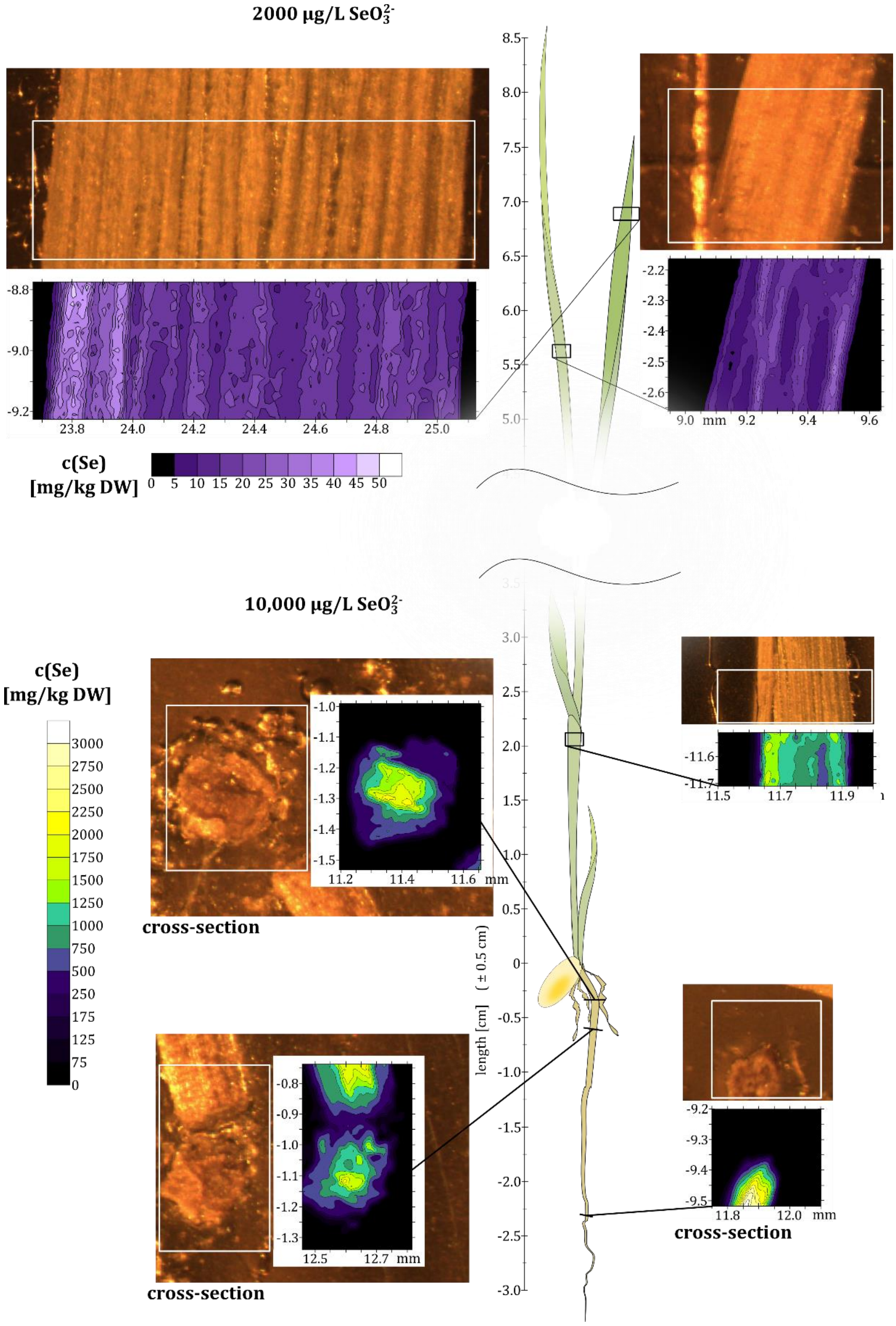

S1 Fig: Selenium fluorescence mappings of shoot and root tissue of a dried rice plant treated with 2000 µg/L Se (top) and 10,000 µg/L Se (bottom) as Na_2SeO_3;photos taken with a binocular
