# Supplementary figures and images for: Tracking Se Assimilation and Speciation through the Rice Plant – Nutrient Competition, Toxicity and Distribution
Source: PLoS One. 2016 Apr 26;11(4):e0152081. doi: 10.1371/journal.pone.0152081 (PMC4846085; doi:10.1371/journal.pone.0152081)

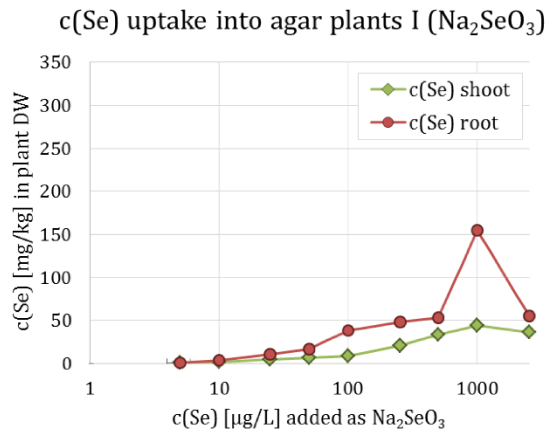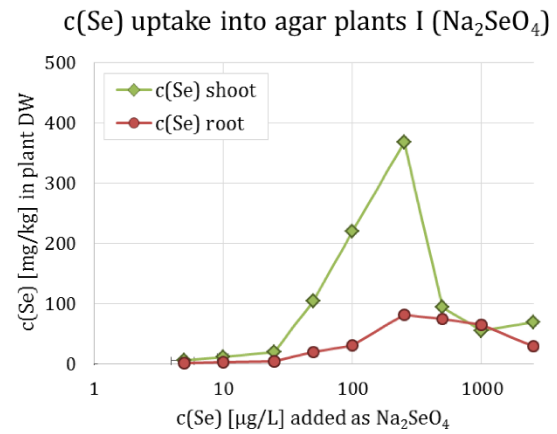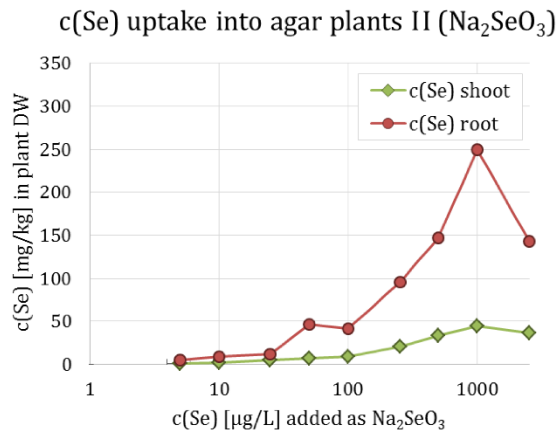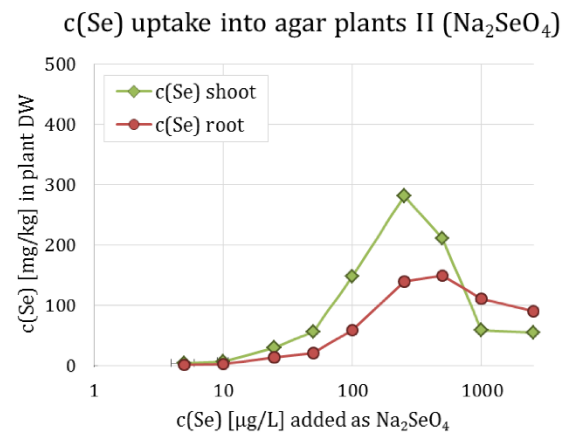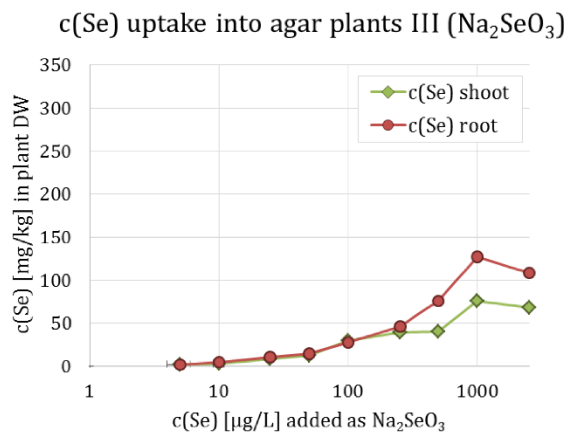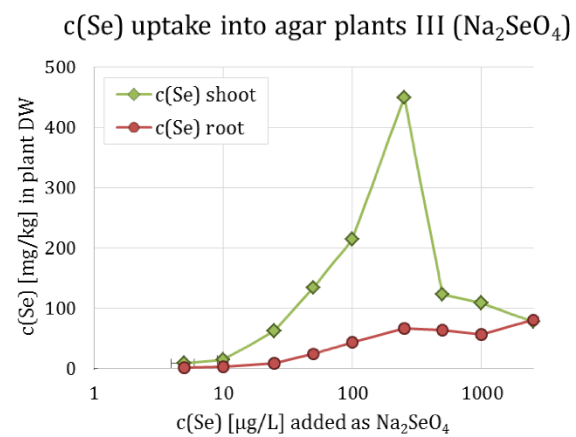

**S1 Fig: Results for plant Se content for each of the three experimental runs of agar experiments**

Supplement: S8 Fig — (PDF) [file pone.0152081.s008.pdf]

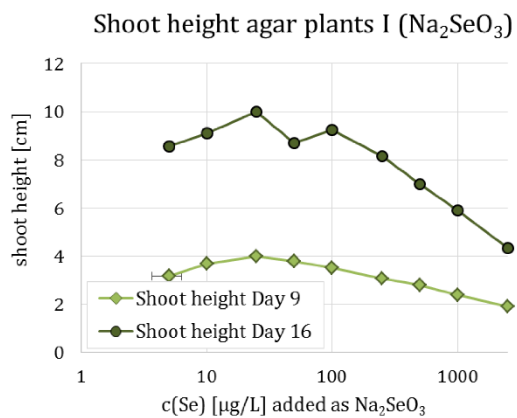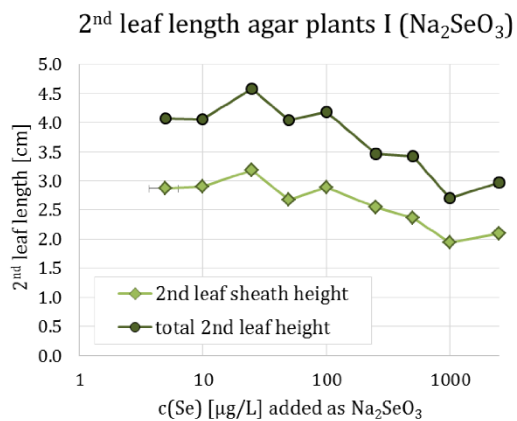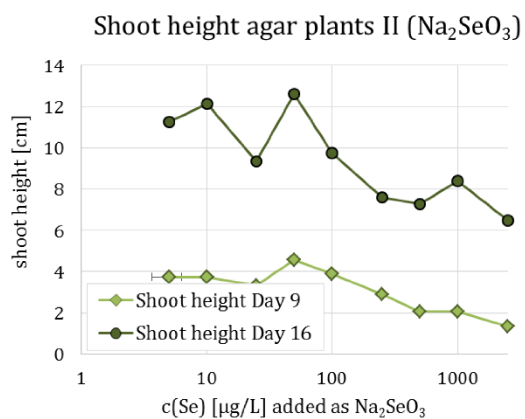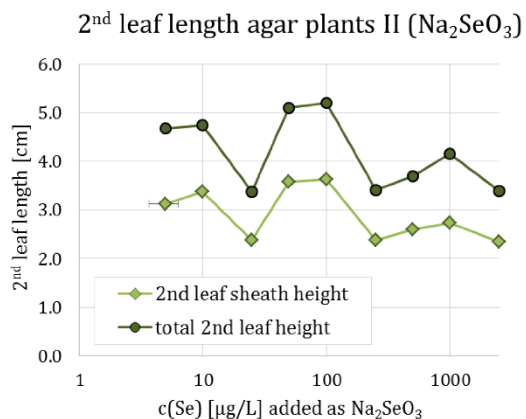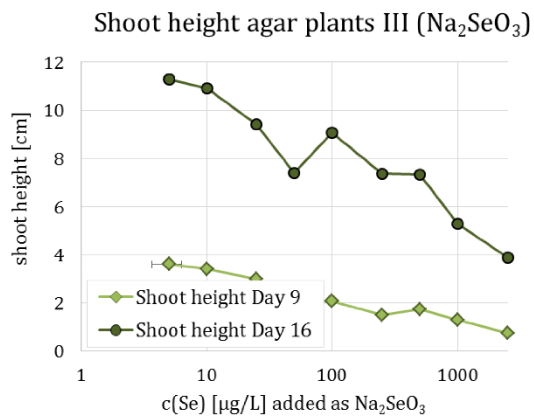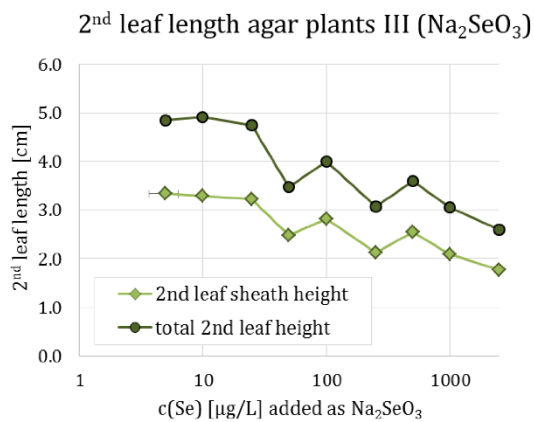

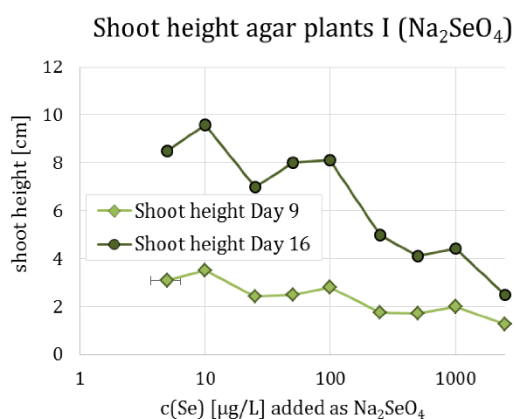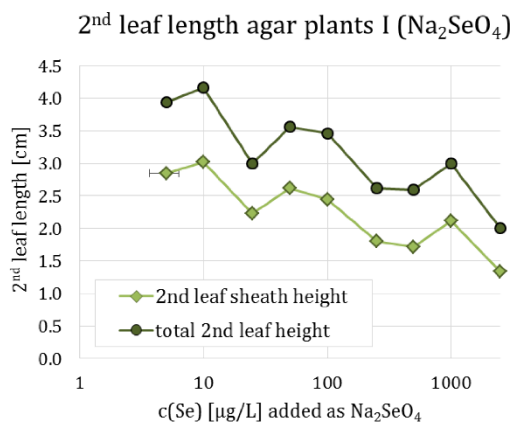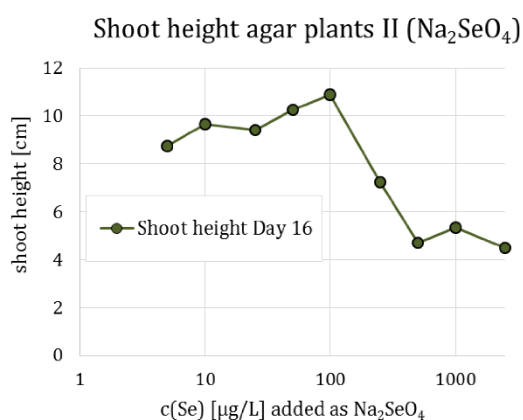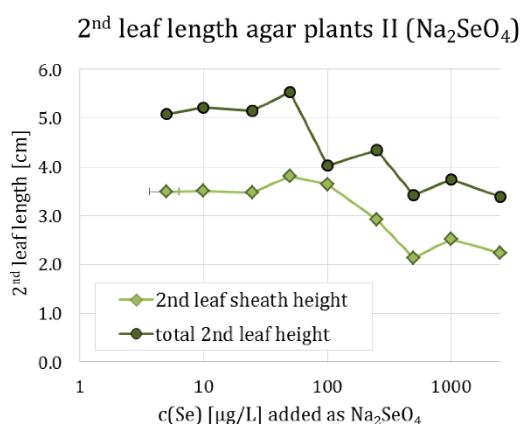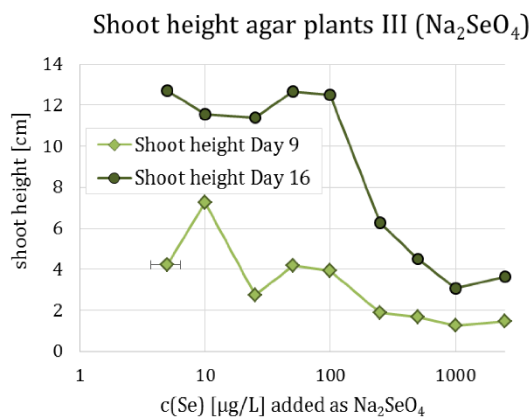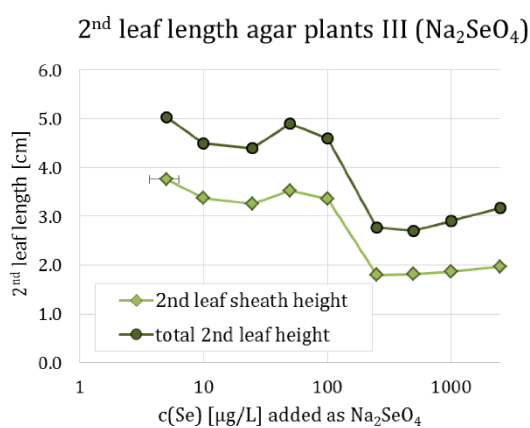

S10 Fig: Results for shoot height and length of the 2nd leaf for plants from the agar experiments

Supplement: S10 Fig — (PDF) [file pone.0152081.s010.pdf]

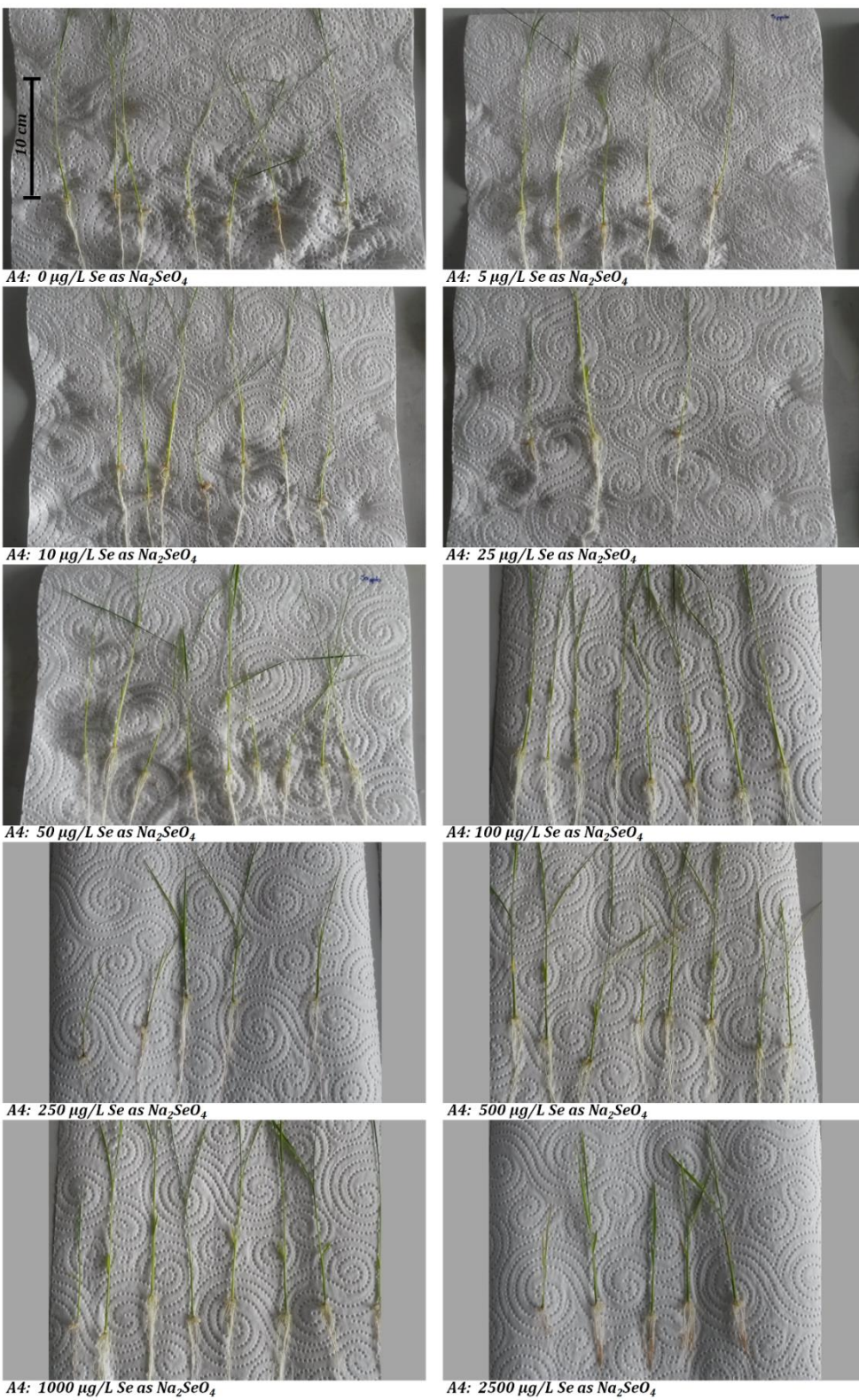

**S18 Fig: Photos of harvested plants treated with  $\text{Na}_2\text{SeO}_4$  in phytoagar & delayed Se**

Supplement: S18 Fig — (PDF) [file pone.0152081.s018.pdf]
