# Supplementary material for: Tracking Se Assimilation and Speciation through the Rice Plant – Nutrient Competition, Toxicity and Distribution
Source: PLoS One. 2016 Apr 26;11(4):e0152081. doi: 10.1371/journal.pone.0152081 (PMC4846085; doi:10.1371/journal.pone.0152081)
Supplement: S9 Fig — (PDF) [file pone.0152081.s009.pdf]

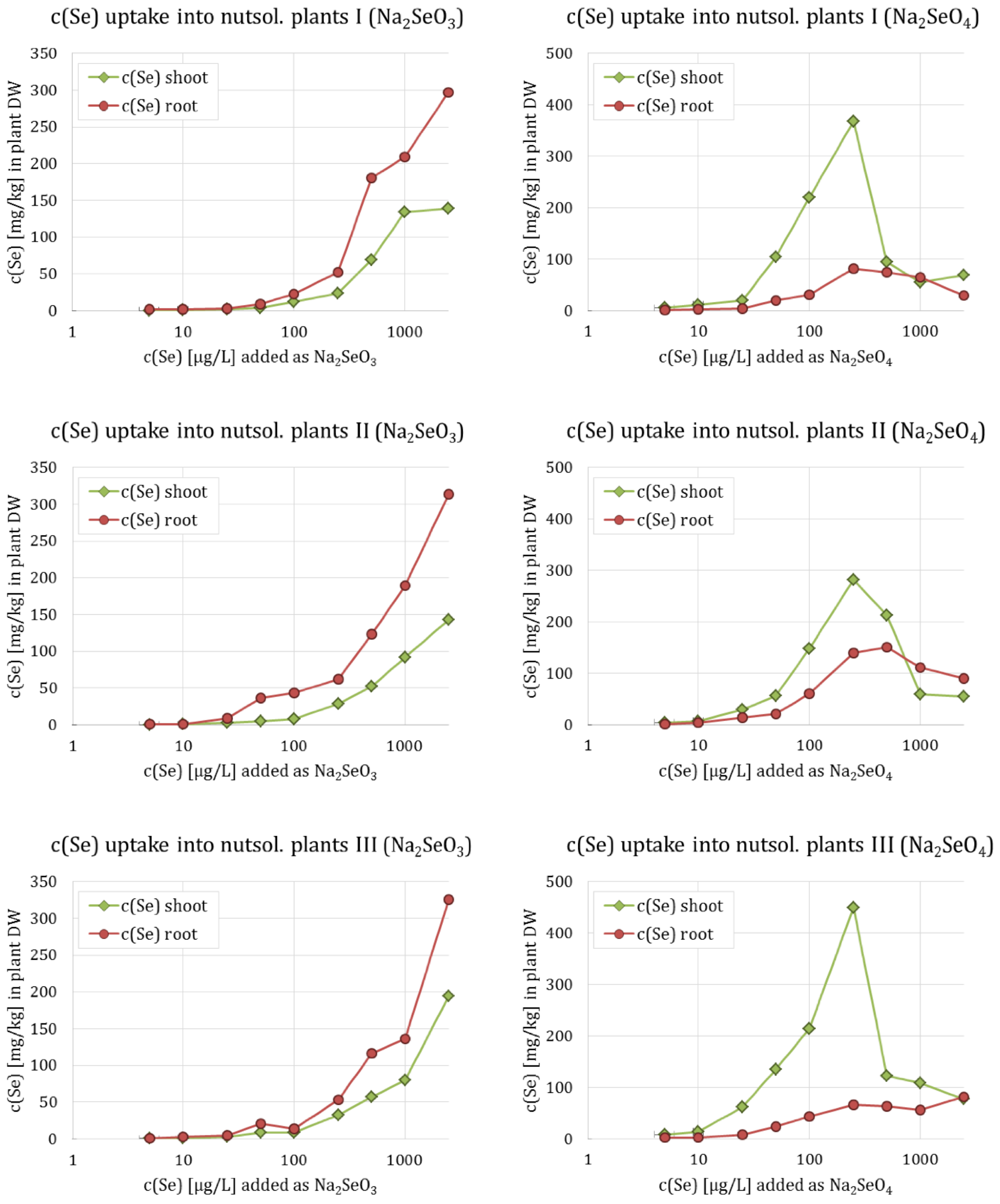


**S1 Fig: Results for plant Se content for each of the three experimental runs of nutrient solution experiments**
