# Supplementary material for: Tracking Se Assimilation and Speciation through the Rice Plant – Nutrient Competition, Toxicity and Distribution
Source: PLoS One. 2016 Apr 26;11(4):e0152081. doi: 10.1371/journal.pone.0152081 (PMC4846085; doi:10.1371/journal.pone.0152081)
Supplement: S11 Fig — (PDF) [file pone.0152081.s011.pdf]

Shoot height nut.sol. plants I (Na_2SeO_3)

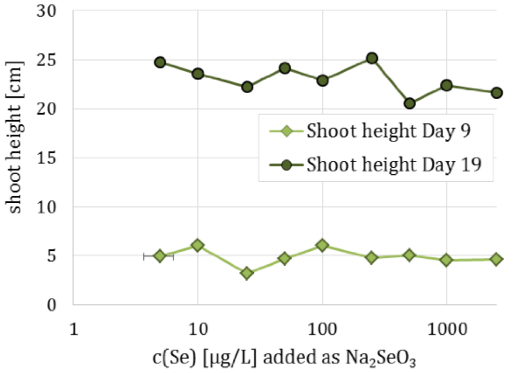

2nd leaf nut.sol. plants I (Na_2SeO_3)

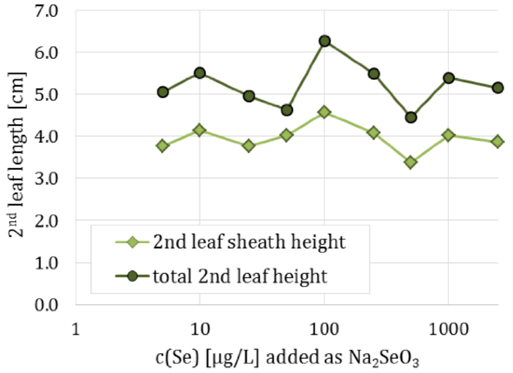

Shoot height nut.sol. plants II (Na_2SeO_3)

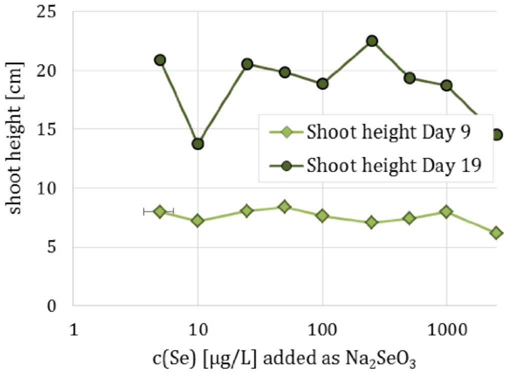

2nd leaf nut.sol. plants II (Na_2SeO_3)

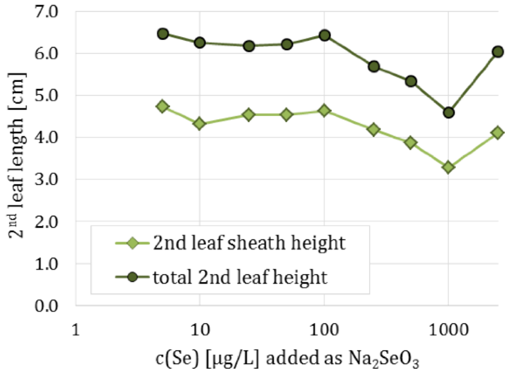

Shoot height nut.sol. plants III (Na_2SeO_3)

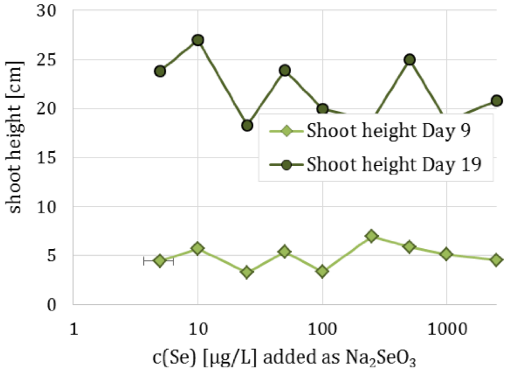

2nd leaf nut.sol. plants III (Na_2SeO_3)

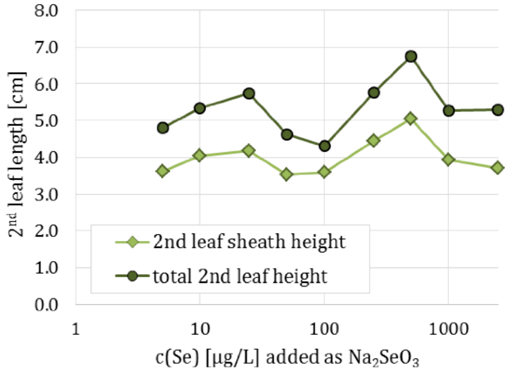

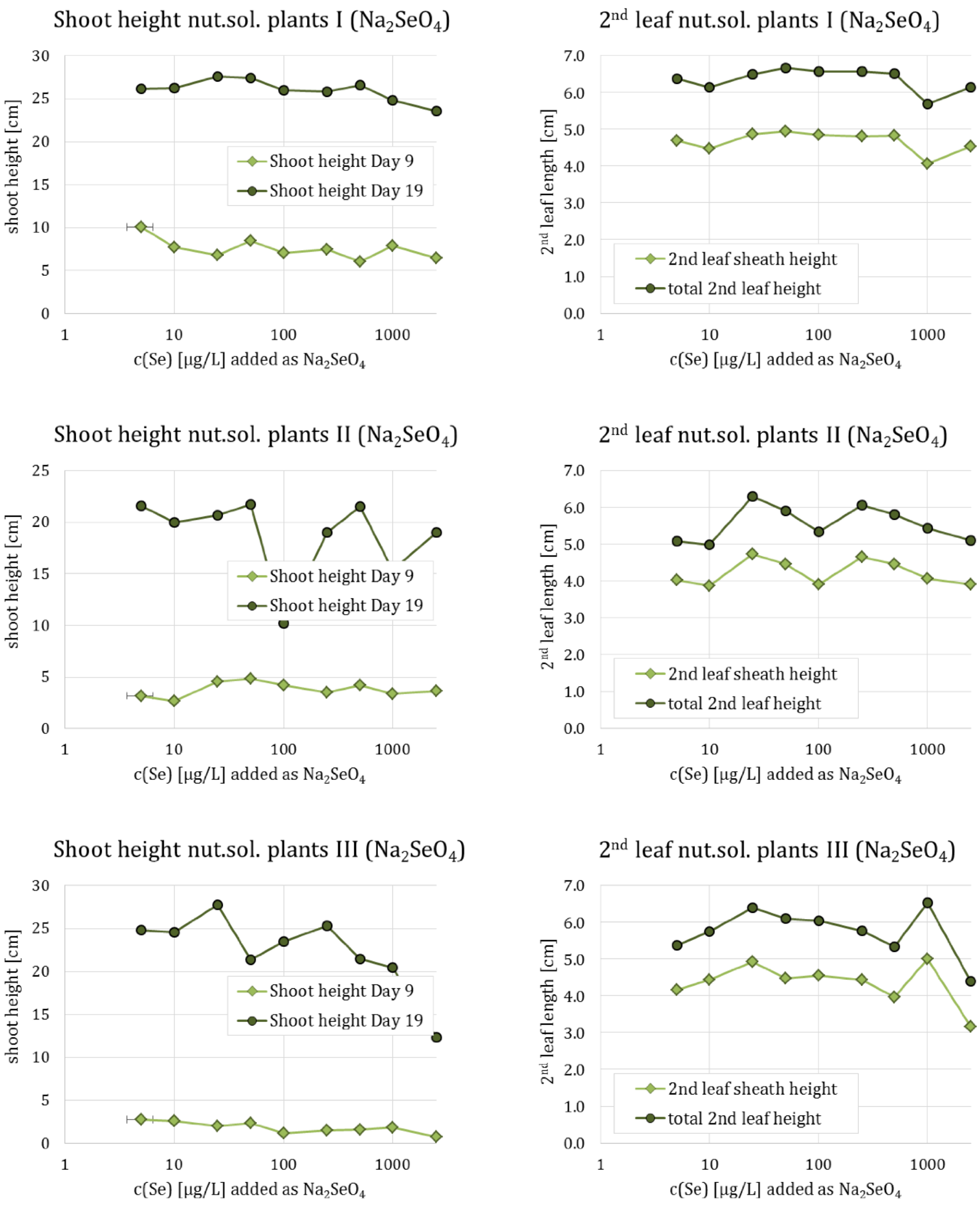

S11 Fig: Results for shoot height and length of the 2nd leaf for plants from the nutrient solution experiments
