# Supplementary material for: Tracking Se Assimilation and Speciation through the Rice Plant – Nutrient Competition, Toxicity and Distribution
Source: PLoS One. 2016 Apr 26;11(4):e0152081. doi: 10.1371/journal.pone.0152081 (PMC4846085; doi:10.1371/journal.pone.0152081)
Supplement: S12 Fig — (PDF) [file pone.0152081.s012.pdf]

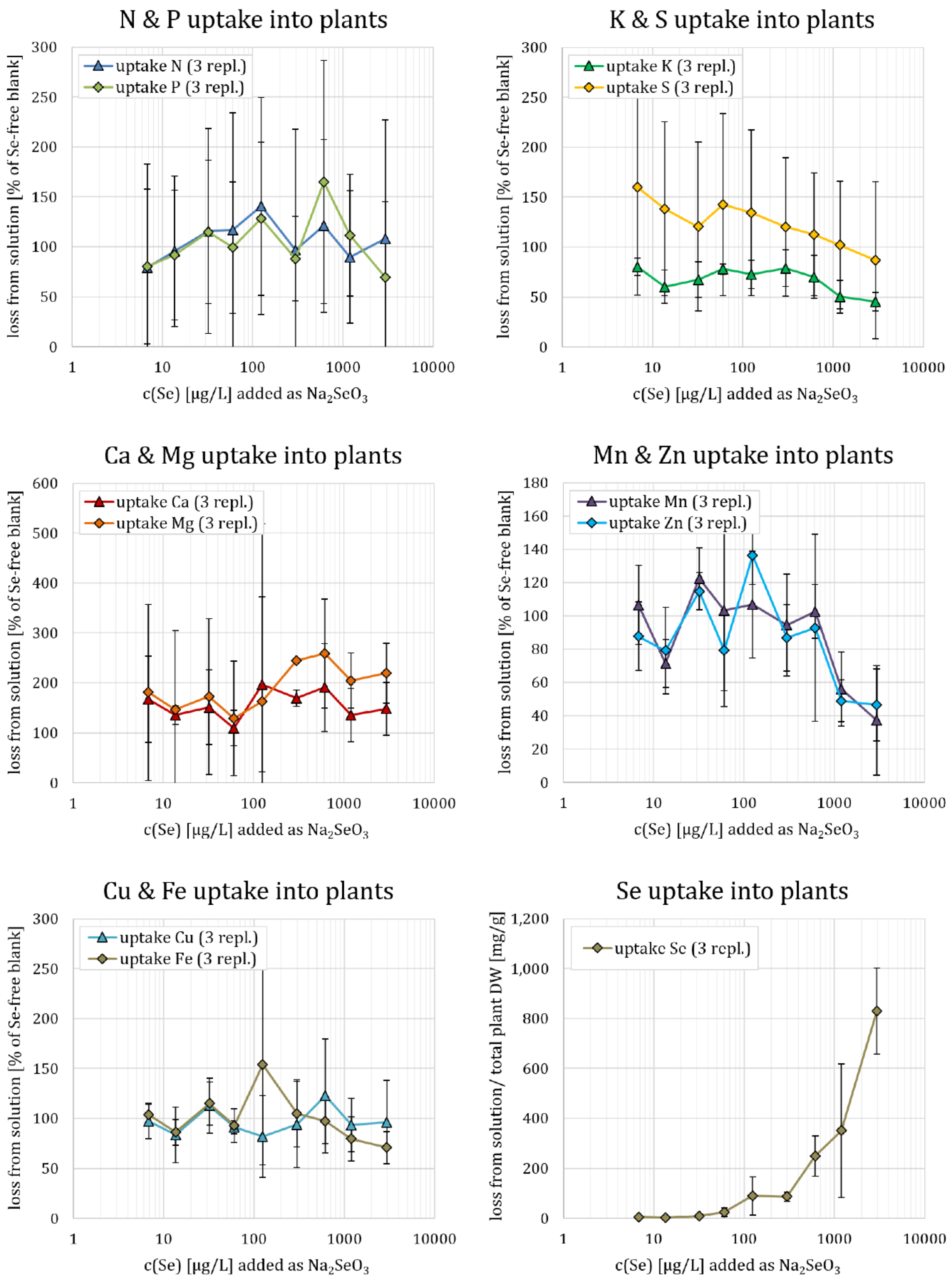
N & P uptake into plants
uptake N (3 repl.)
uptake P (3 repl.)
loss from solution [% of Se-free blank]
c(Se) [µg/L] added as Na_2SeO_3
K & S uptake into plants
uptake K (3 repl.)
uptake S (3 repl.)
loss from solution [% of Se-free blank]
c(Se) [µg/L] added as Na_2SeO_3
Ca & Mg uptake into plants
uptake Ca (3 repl.)
uptake Mg (3 repl.)
loss from solution [% of Se-free blank]
c(Se) [µg/L] added as Na_2SeO_3
Mn & Zn uptake into plants
uptake Mn (3 repl.)
uptake Zn (3 repl.)
loss from solution [% of Se-free blank]
c(Se) [µg/L] added as Na_2SeO_3
Cu & Fe uptake into plants
uptake Cu (3 repl.)
uptake Fe (3 repl.)
loss from solution [% of Se-free blank]
c(Se) [µg/L] added as Na_2SeO_3
Se uptake into plants
uptake Se (3 repl.)
loss from solution/ total plant DW [mg/g]
c(Se) [µg/L] added as Na_2SeO_3

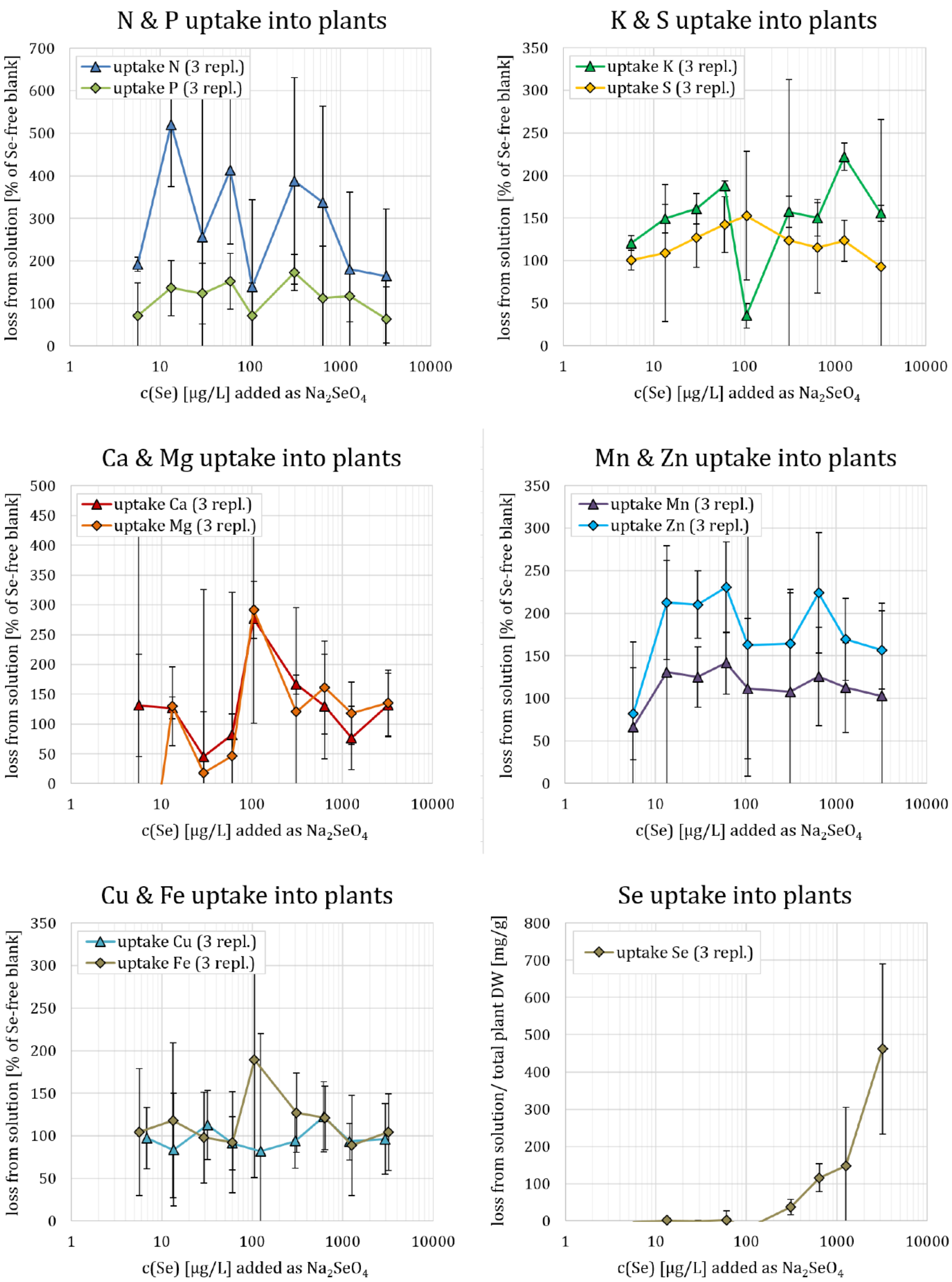

S12 Fig: Calculated element uptake into plants from nutrient solution plotted against added Se concentration
