# Supplementary material for: Tracking Se Assimilation and Speciation through the Rice Plant – Nutrient Competition, Toxicity and Distribution
Source: PLoS One. 2016 Apr 26;11(4):e0152081. doi: 10.1371/journal.pone.0152081 (PMC4846085; doi:10.1371/journal.pone.0152081)
Supplement: S14 Fig — (PDF) [file pone.0152081.s014.pdf]

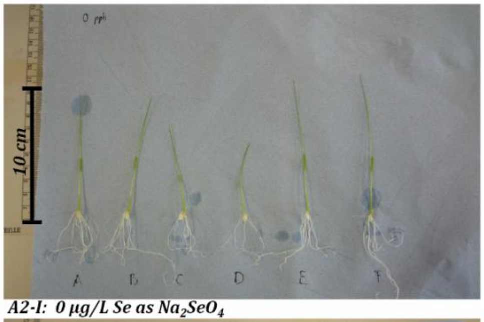

A2-I: 0 μg/L Se as Na_2SeO_4

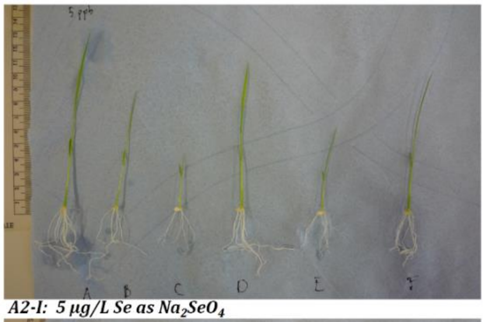
A2-I: 5 μg/L Se as Na_2SeO_4

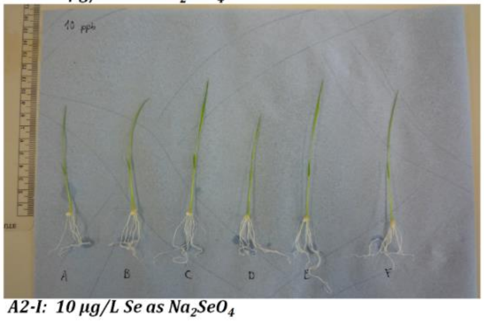
A2-I: 10 μg/L Se as Na_2SeO_4

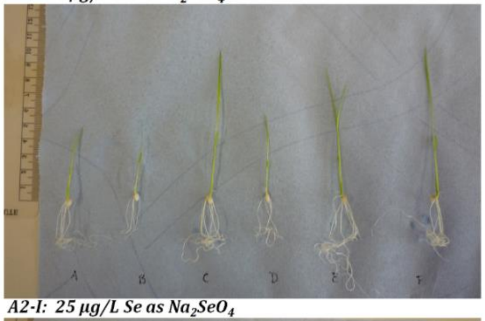
A2-I: 25 μg/L Se as Na_2SeO_4

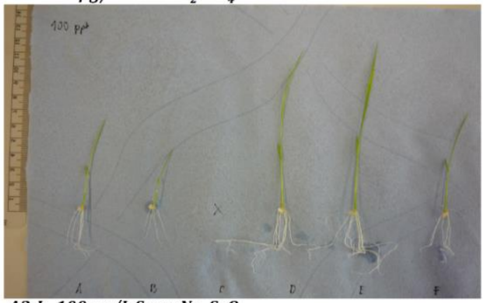
A2-I: 100 μg/L Se as Na_2SeO_4

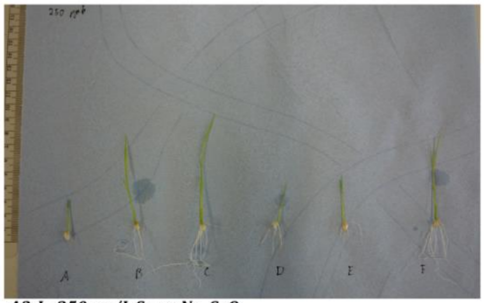
A2-I: 250 μg/L Se as Na_2SeO_4

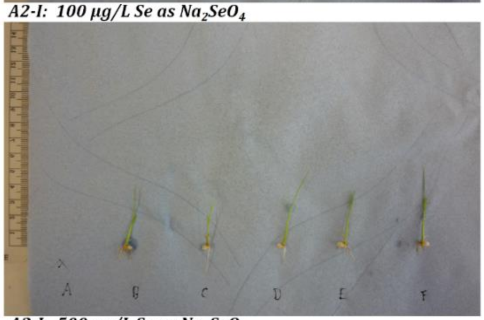
A2-I: 500 μg/L Se as Na_2SeO_4

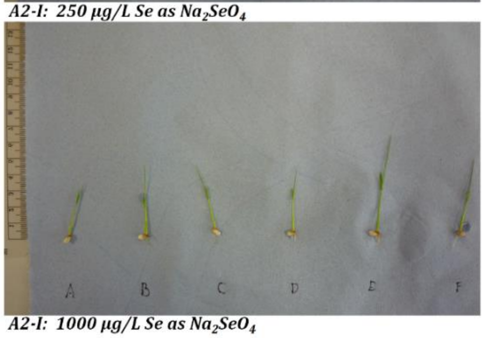
A2-I: 1000 μg/L Se as Na_2SeO_4

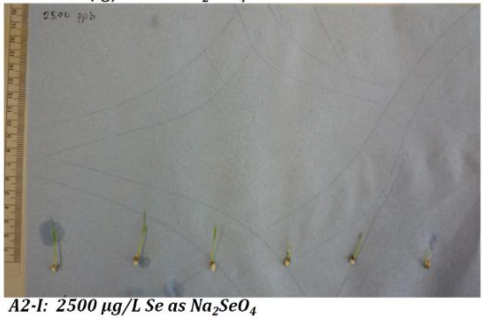
A2-I: 2500 μg/L Se as Na_2SeO_4

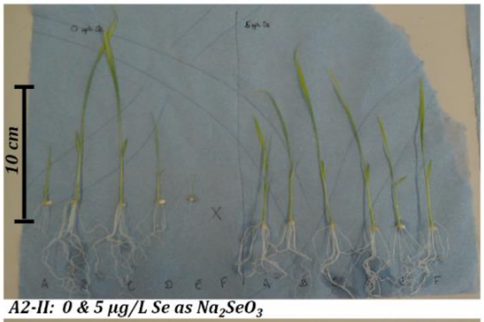

A2-II: 0 & 5 μg/L Se as Na_2SeO_3

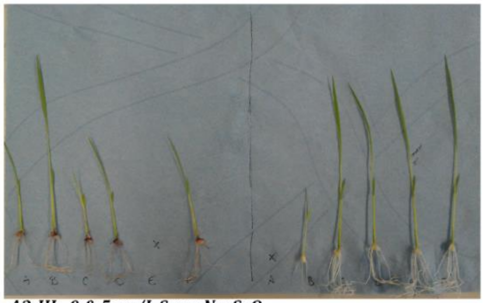

A2-III: 0 & 5 μg/L Se as Na_2SeO_3

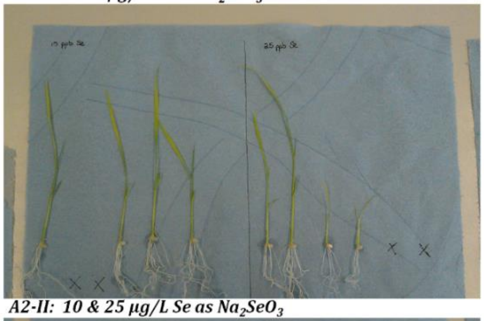

A2-II: 10 & 25 μg/L Se as Na_2SeO_3

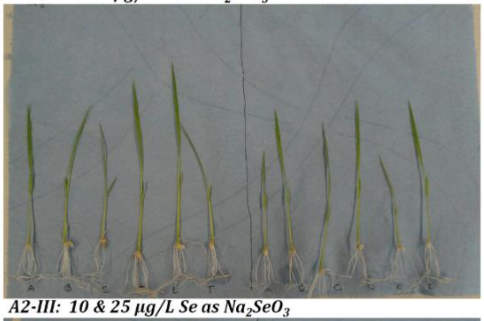

A2-III: 10 & 25 μg/L Se as Na_2SeO_3

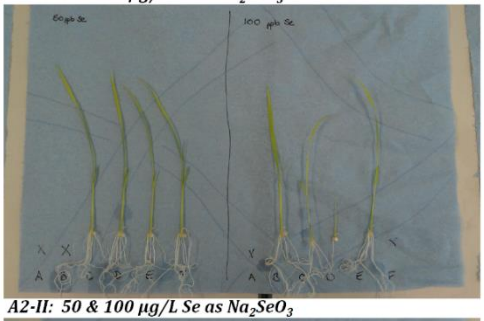

A2-II: 50 & 100 μg/L Se as Na_2SeO_3

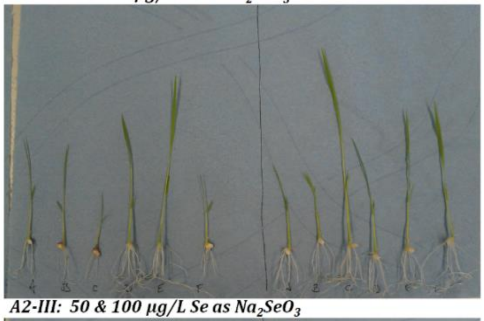

A2-III: 50 & 100 μg/L Se as Na_2SeO_3

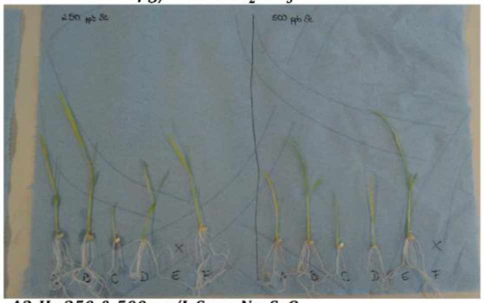

A2-II: 250 & 500 μg/L Se as Na_2SeO_3

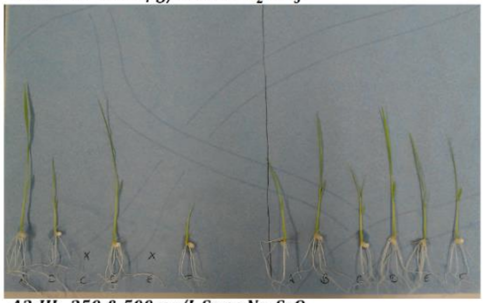

A2-III: 250 & 500 μg/L Se as Na_2SeO_3

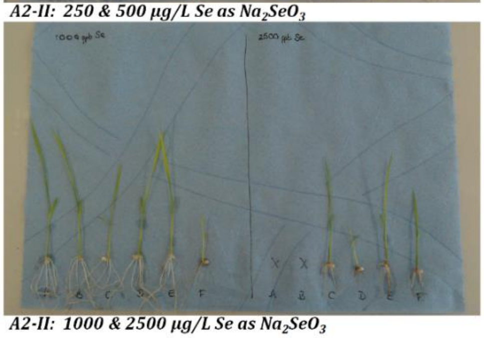

A2-II: 1000 & 2500 μg/L Se as Na_2SeO_3

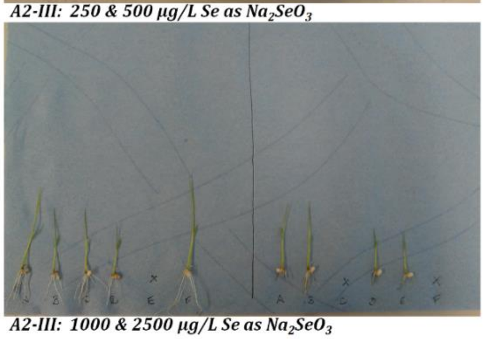

A2-III: 1000 & 2500 μg/L Se as Na_2SeO_3

S14 Fig: Photos of harvested plants treated with Na_2SeO_4 in phytoagar & direct Se
