# Supplementary material for: Tracking Se Assimilation and Speciation through the Rice Plant – Nutrient Competition, Toxicity and Distribution
Source: PLoS One. 2016 Apr 26;11(4):e0152081. doi: 10.1371/journal.pone.0152081 (PMC4846085; doi:10.1371/journal.pone.0152081)
Supplement: S17 Fig — (PDF) [file pone.0152081.s017.pdf]

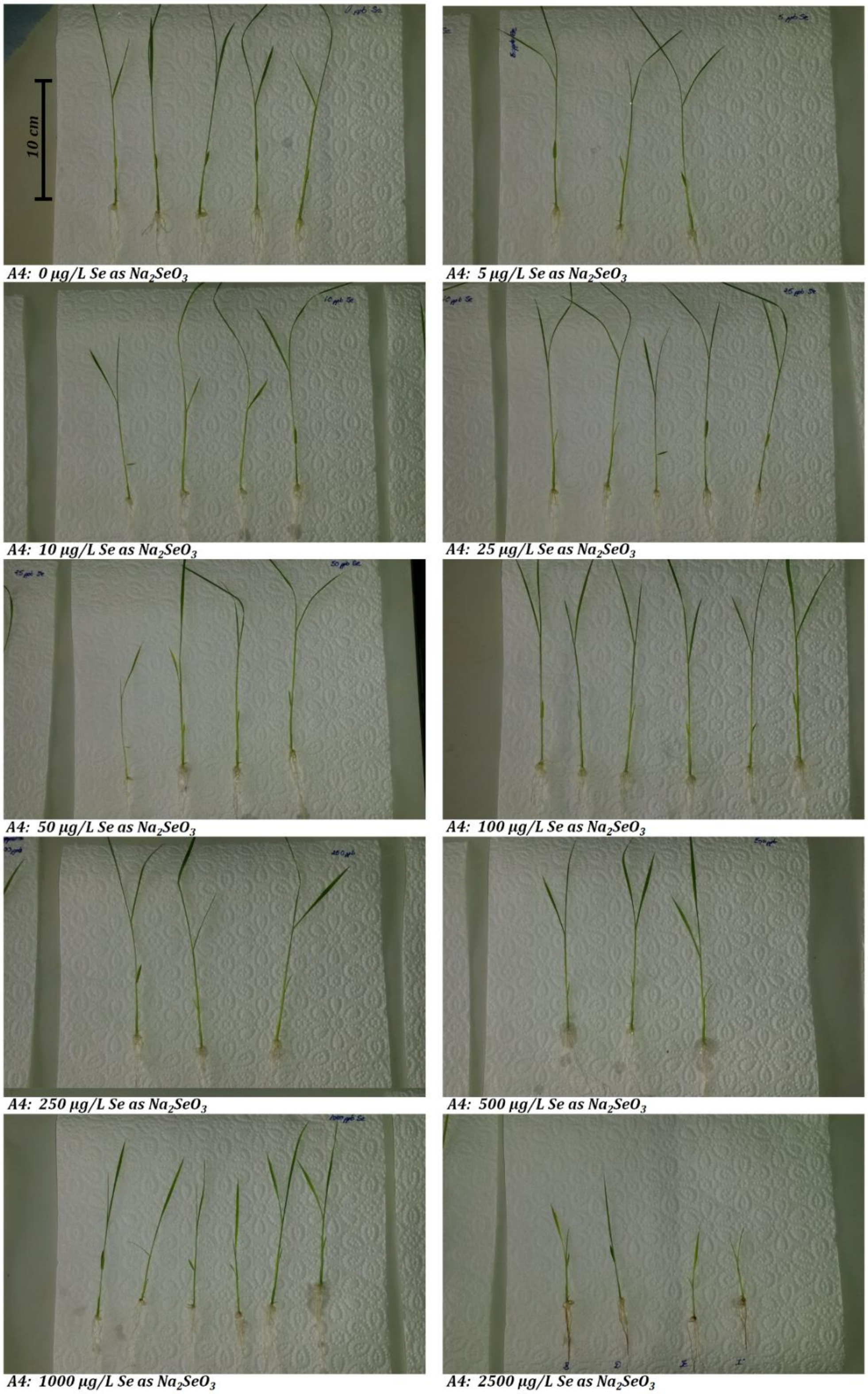

S17 Fig: Photos of harvested plants treated with Na_2SeO_3 in phytoagar & delayed Se
