# Supplementary material for: Tracking Se Assimilation and Speciation through the Rice Plant – Nutrient Competition, Toxicity and Distribution
Source: PLoS One. 2016 Apr 26;11(4):e0152081. doi: 10.1371/journal.pone.0152081 (PMC4846085; doi:10.1371/journal.pone.0152081)
Supplement: S19 Fig — (PDF) [file pone.0152081.s019.pdf]

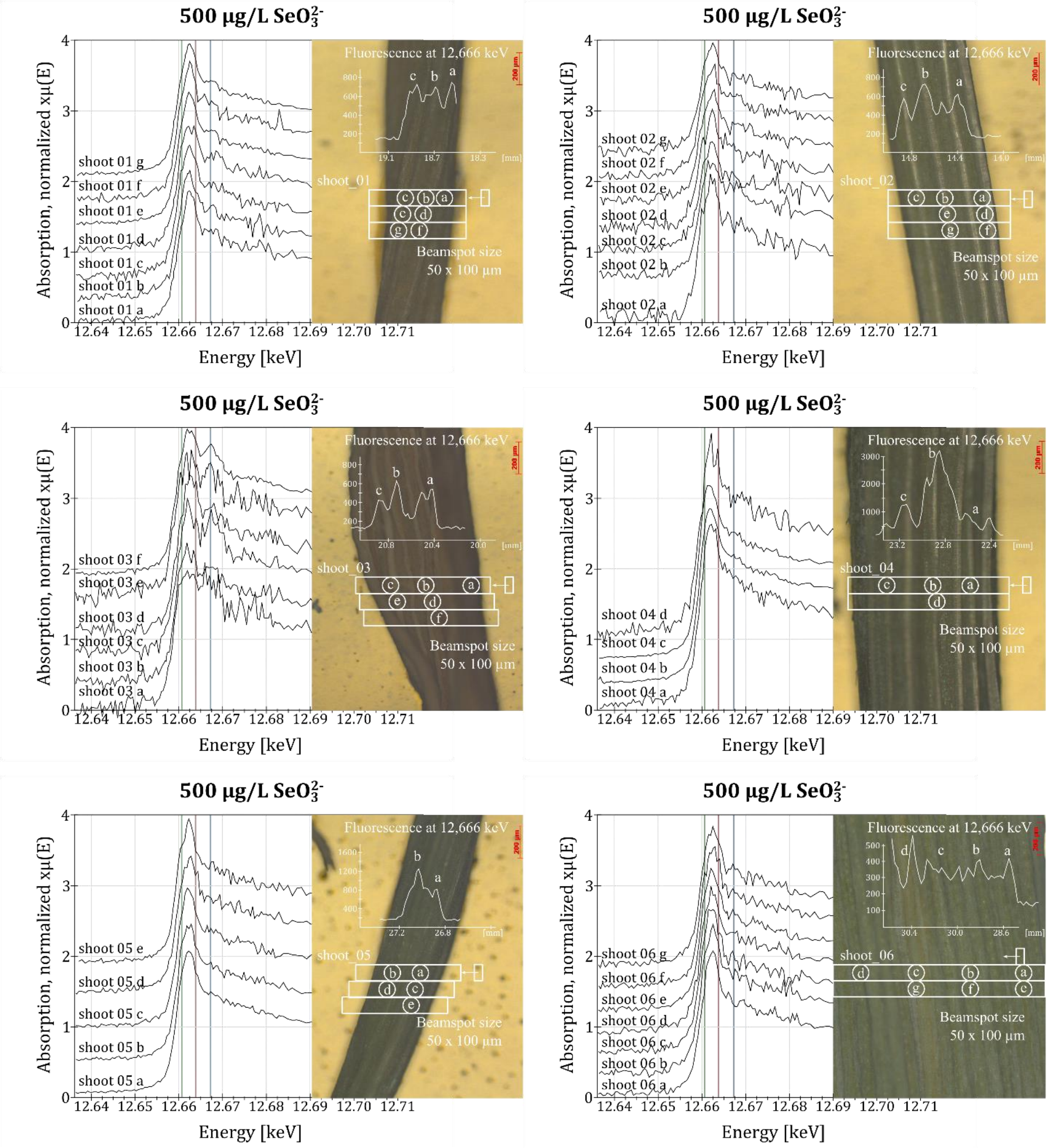

500 µg/L SeO3 2-
Absorption, normalized xµ(E)
Energy [keV]
12.64 12.65 12.66 12.67 12.68 12.69 12.70 12.71
Fluorescence at 12,666 keV
[mm]
shoot 01 g
shoot 01 f
shoot 01 e
shoot 01 d
shoot 01 c
shoot 01 b
shoot 01 a
shoot_01
shoot 02 g
shoot 02 f
shoot 02 e
shoot 02 d
shoot 02 c
shoot 02 b
shoot 02 a
shoot_02
shoot 03 f
shoot 03 e
shoot 03 d
shoot 03 c
shoot 03 b
shoot 03 a
shoot_03
shoot 04 d
shoot 04 c
shoot 04 b
shoot 04 a
shoot_04
shoot 05 e
shoot 05 d
shoot 05 c
shoot 05 b
shoot 05 a
shoot_05
shoot 06 g
shoot 06 f
shoot 06 e
shoot 06 d
shoot 06 c
shoot 06 b
shoot 06 a
shoot_06
Beamspot size
50 x 100 µm

500 µg/L SeO_3^{2-}

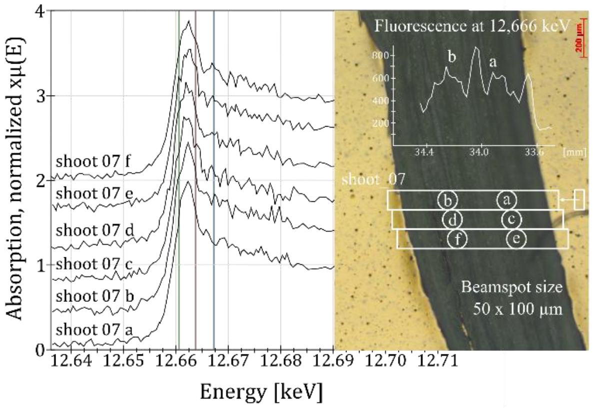

500 µg/L SeO_3^{2-}

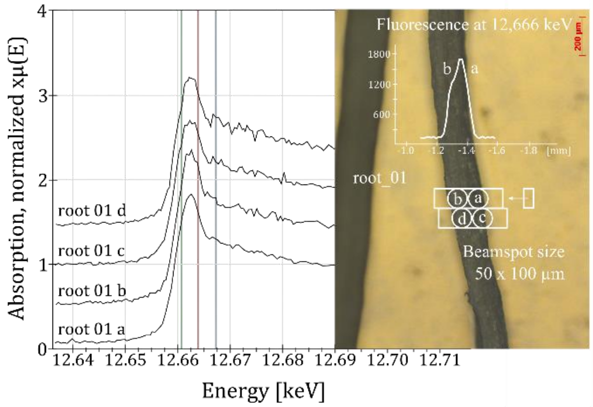

500 µg/L SeO_3^{2-}

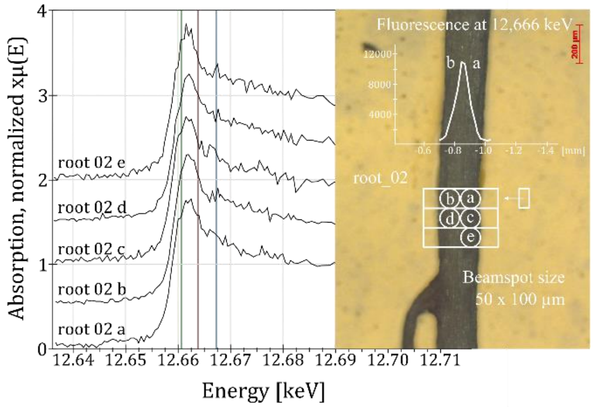

500 µg/L SeO_3^{2-}

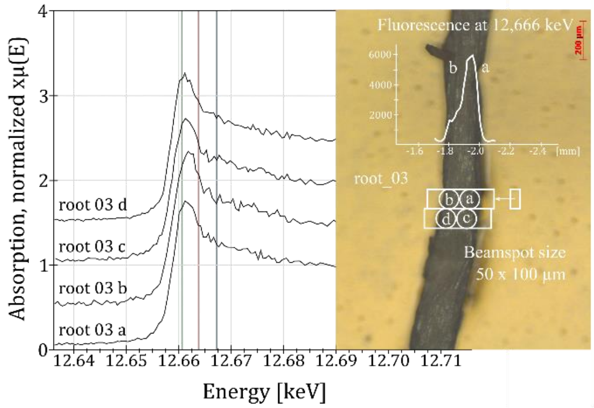

500 µg/L SeO_3^{2-}

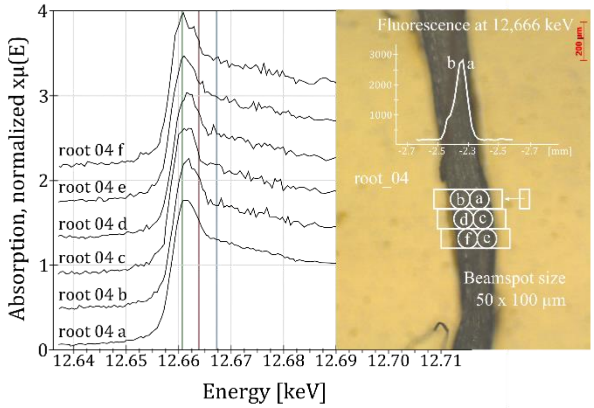

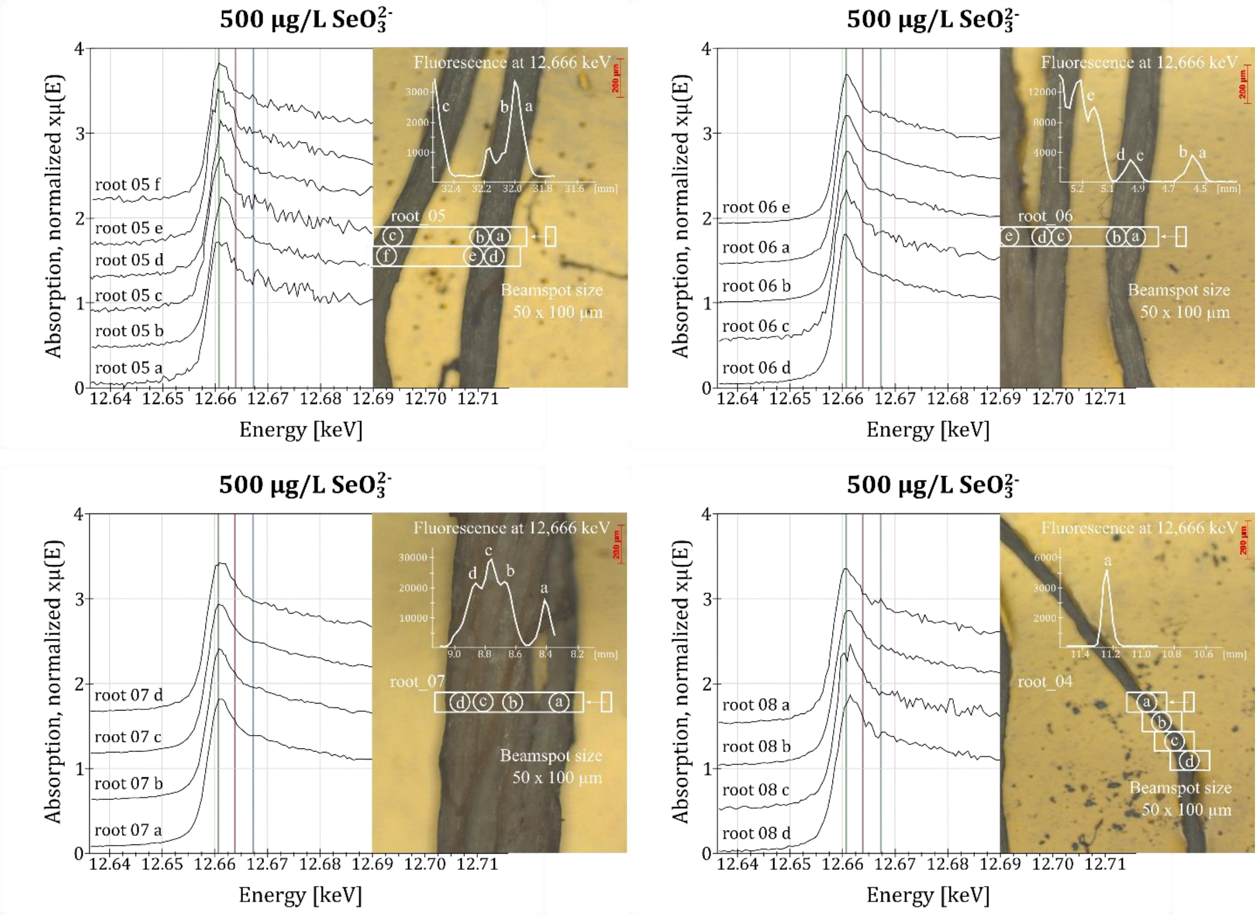

S1 Fig: all XANES spectra of each region of interest (ROI) on a rice plant treated with 500 µg/L Se as Na_2SeO_3 in nutrient solution
