# Supplementary material for: Tracking Se Assimilation and Speciation through the Rice Plant – Nutrient Competition, Toxicity and Distribution
Source: PLoS One. 2016 Apr 26;11(4):e0152081. doi: 10.1371/journal.pone.0152081 (PMC4846085; doi:10.1371/journal.pone.0152081)
Supplement: S20 Fig — (PDF) [file pone.0152081.s020.pdf]

500 µg/L SeO_4^{2-}

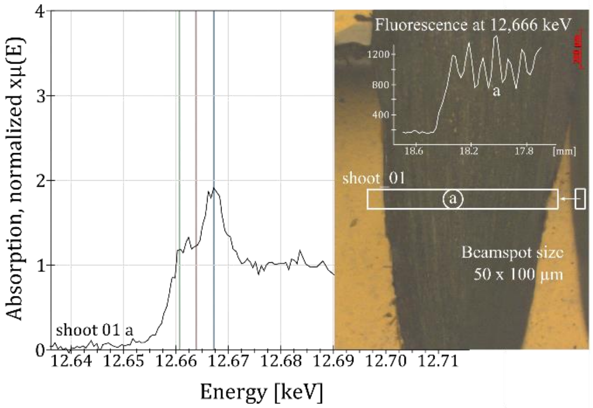

500 µg/L SeO_4^{2-}

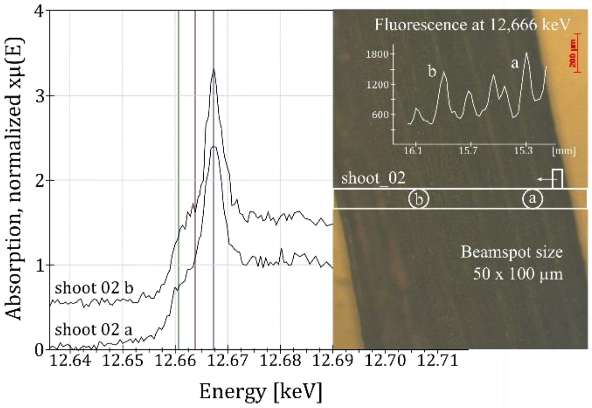

500 µg/L SeO_4^{2-}

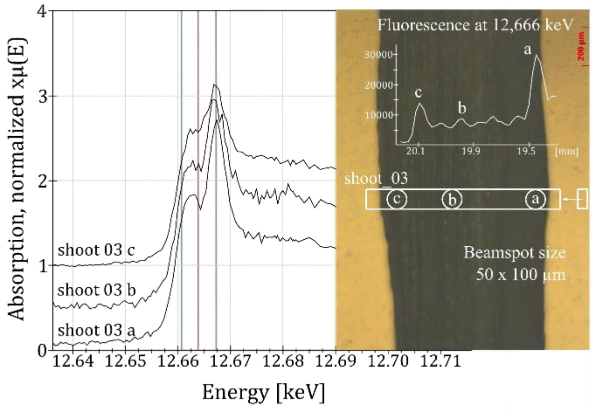

500 µg/L SeO_4^{2-}

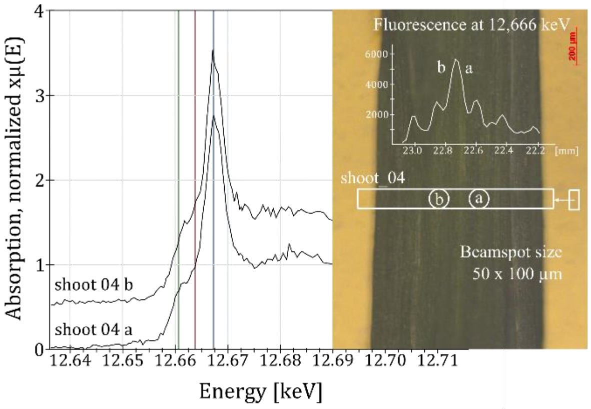

500 µg/L SeO_4^{2-}

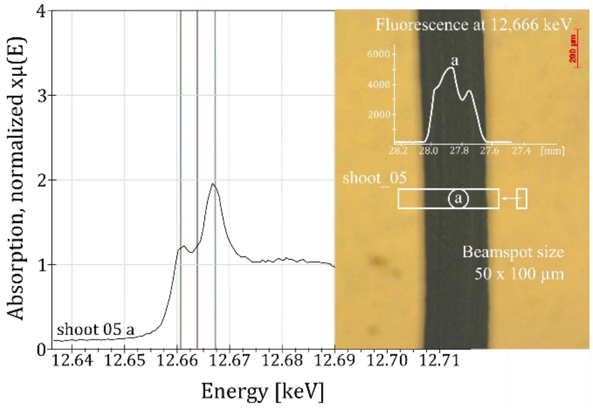

500 µg/L SeO_4^{2-}

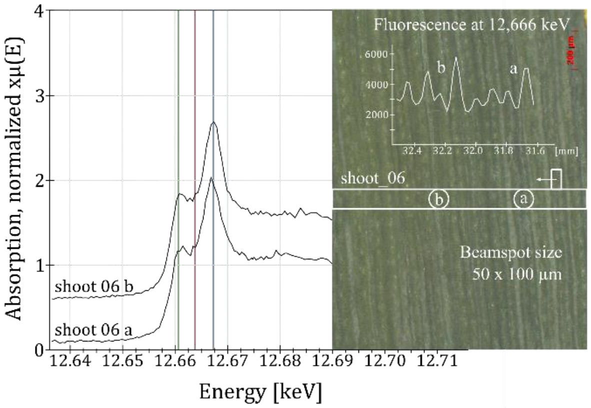

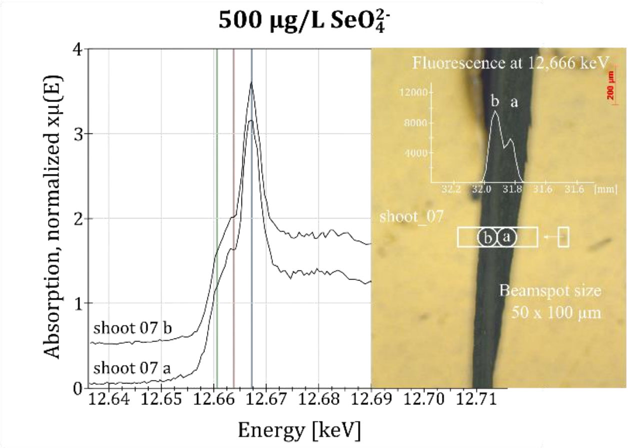
500 µg/L SeO$_4^{2-}$
Absorption, normalized xµ(E)
Energy [keV]
12.64 12.65 12.66 12.67 12.68 12.69 12.70 12.71
shoot 07 b
shoot 07 a
Fluorescence at 12,666 keV
shoot_07
Beamspot size
50 x 100 µm

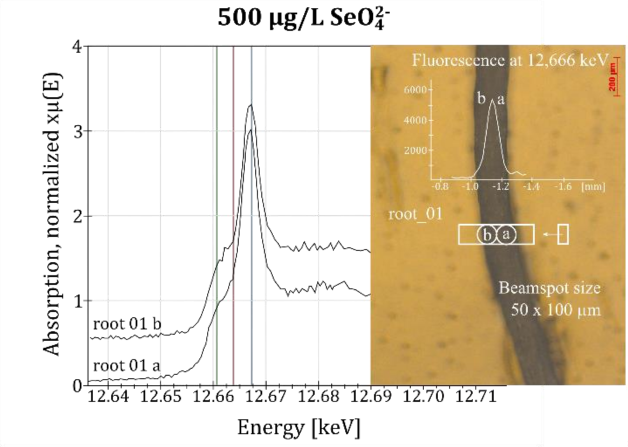
500 µg/L SeO$_4^{2-}$
Absorption, normalized xµ(E)
Energy [keV]
12.64 12.65 12.66 12.67 12.68 12.69 12.70 12.71
root 01 b
root 01 a
Fluorescence at 12,666 keV
root_01
Beamspot size
50 x 100 µm

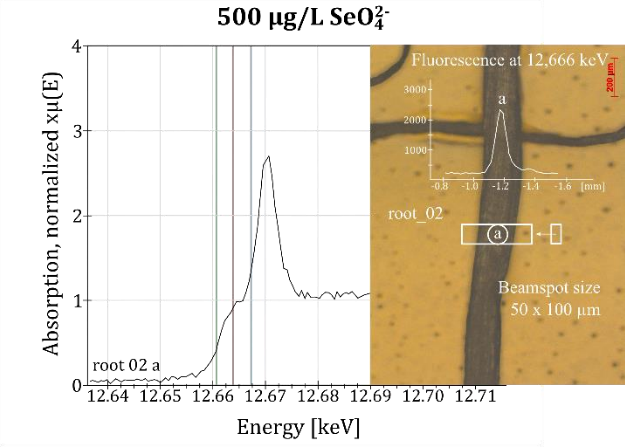
500 µg/L SeO$_4^{2-}$
Absorption, normalized xµ(E)
Energy [keV]
12.64 12.65 12.66 12.67 12.68 12.69 12.70 12.71
root 02 a
Fluorescence at 12,666 keV
root_02
Beamspot size
50 x 100 µm

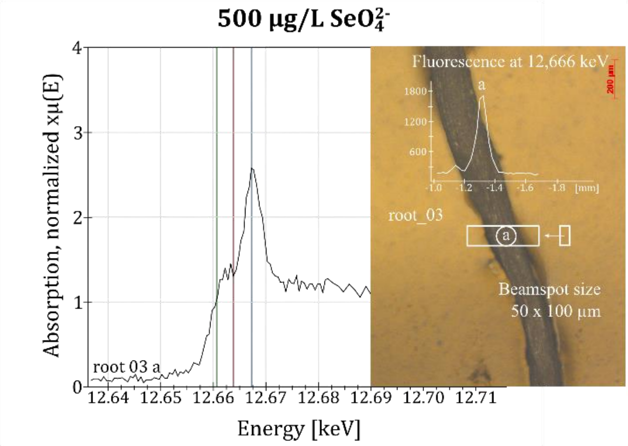
500 µg/L SeO$_4^{2-}$
Absorption, normalized xµ(E)
Energy [keV]
12.64 12.65 12.66 12.67 12.68 12.69 12.70 12.71
root 03 a
Fluorescence at 12,666 keV
root_03
Beamspot size
50 x 100 µm

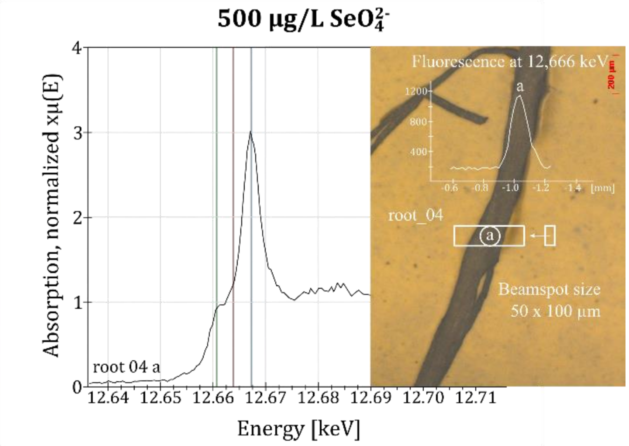
500 µg/L SeO$_4^{2-}$
Absorption, normalized xµ(E)
Energy [keV]
12.64 12.65 12.66 12.67 12.68 12.69 12.70 12.71
root 04 a
Fluorescence at 12,666 keV
root_04
Beamspot size
50 x 100 µm

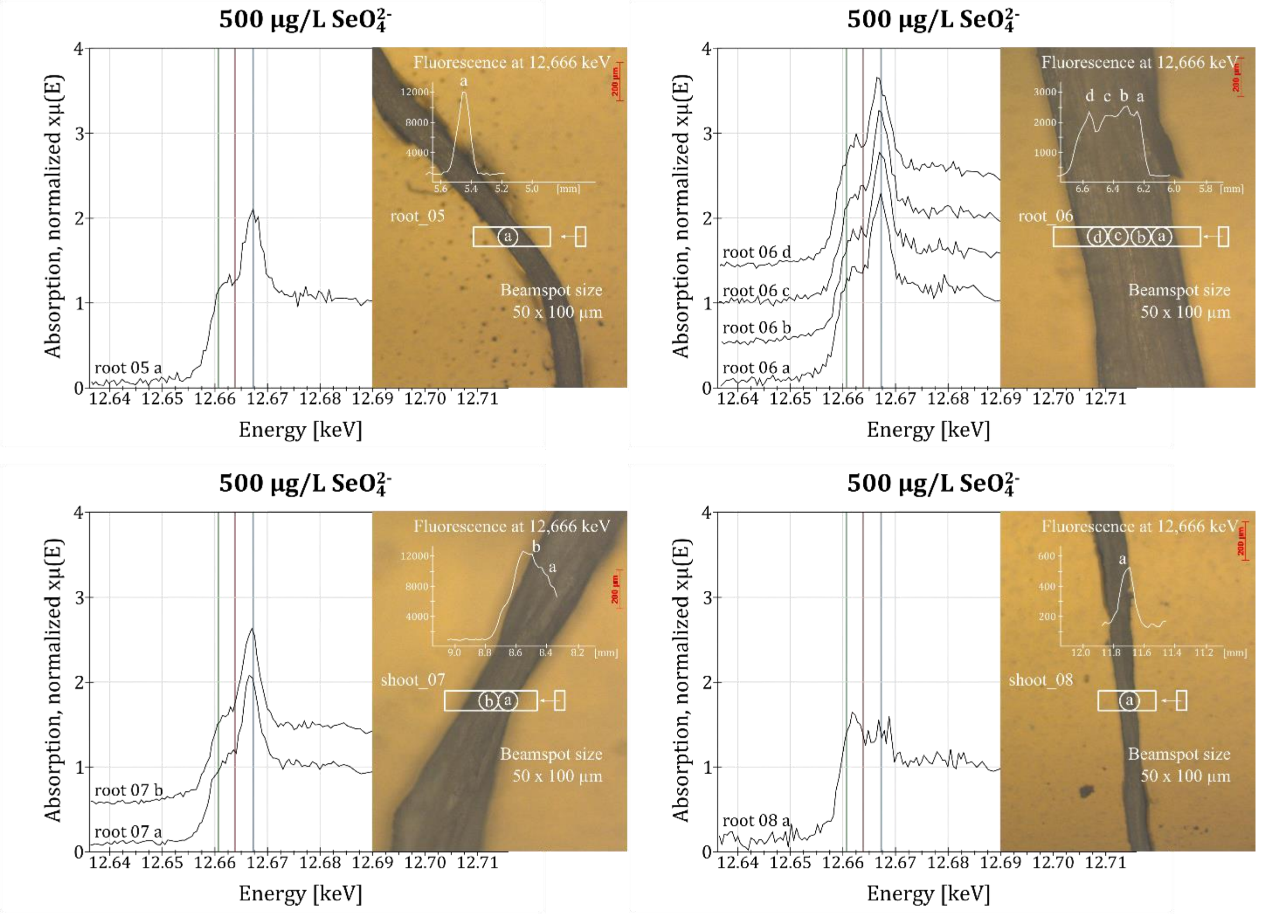

S10 Fig: all XANES spectra of each region of interest (ROI) on a rice plant treated with 500 µg/L Se as Na_2SeO_4 in nutrient solution
