# Supplementary material for: Tracking Se Assimilation and Speciation through the Rice Plant – Nutrient Competition, Toxicity and Distribution
Source: PLoS One. 2016 Apr 26;11(4):e0152081. doi: 10.1371/journal.pone.0152081 (PMC4846085; doi:10.1371/journal.pone.0152081)
Supplement: S21 Fig — (PDF) [file pone.0152081.s021.pdf]

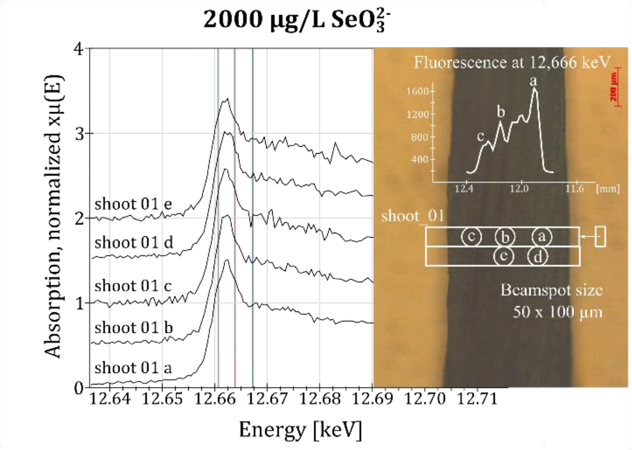
2000 µg/L SeO_3^{2-}
Absorption, normalized xµ(E)
Energy [keV]
shoot 01 e
shoot 01 d
shoot 01 c
shoot 01 b
shoot 01 a
Fluorescence at 12,666 keV
shoot_01
Beamspot size
50 x 100 µm

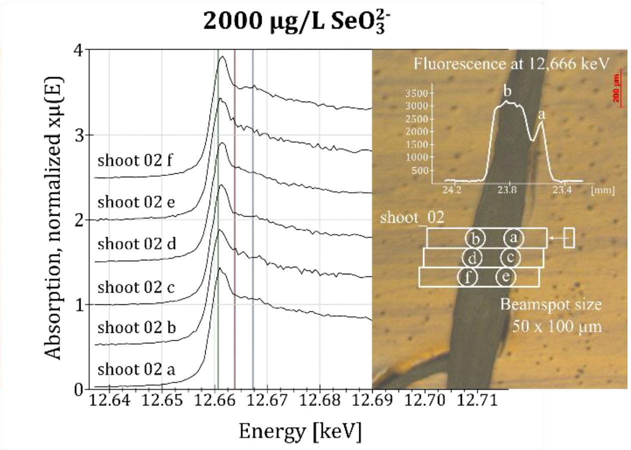
2000 µg/L SeO_3^{2-}
Absorption, normalized xµ(E)
Energy [keV]
shoot 02 f
shoot 02 e
shoot 02 d
shoot 02 c
shoot 02 b
shoot 02 a
Fluorescence at 12,666 keV
shoot_02
Beamspot size
50 x 100 µm

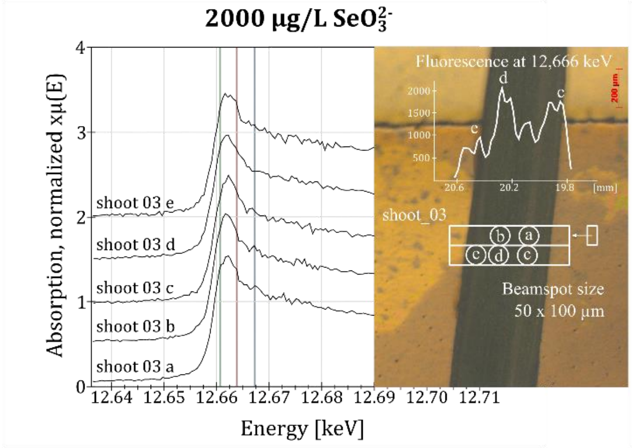
2000 µg/L SeO_3^{2-}
Absorption, normalized xµ(E)
Energy [keV]
shoot 03 e
shoot 03 d
shoot 03 c
shoot 03 b
shoot 03 a
Fluorescence at 12,666 keV
shoot_03
Beamspot size
50 x 100 µm

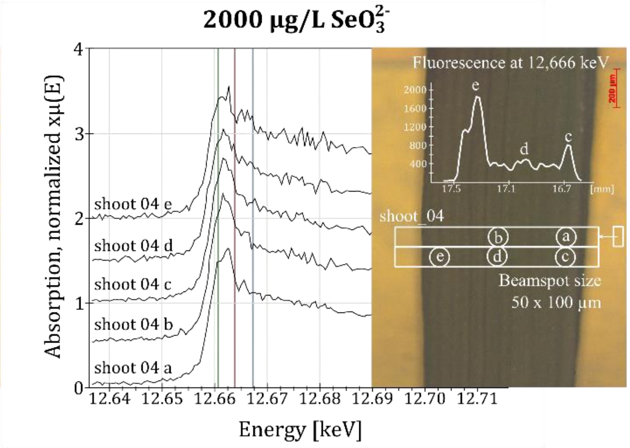
2000 µg/L SeO_3^{2-}
Absorption, normalized xµ(E)
Energy [keV]
shoot 04 e
shoot 04 d
shoot 04 c
shoot 04 b
shoot 04 a
Fluorescence at 12,666 keV
shoot_04
Beamspot size
50 x 100 µm

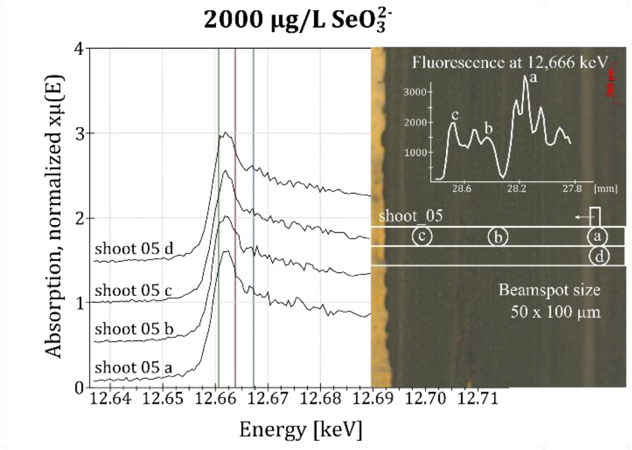
2000 µg/L SeO_3^{2-}
Absorption, normalized xµ(E)
Energy [keV]
shoot 05 d
shoot 05 c
shoot 05 b
shoot 05 a
Fluorescence at 12,666 keV
shoot_05
Beamspot size
50 x 100 µm

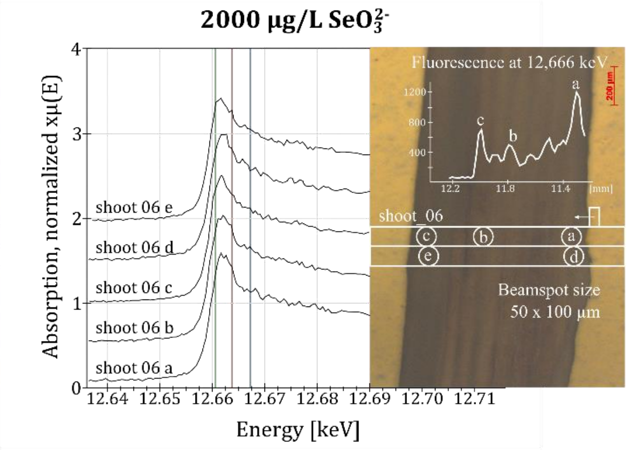
2000 µg/L SeO_3^{2-}
Absorption, normalized xµ(E)
Energy [keV]
shoot 06 e
shoot 06 d
shoot 06 c
shoot 06 b
shoot 06 a
Fluorescence at 12,666 keV
shoot_06
Beamspot size
50 x 100 µm

2000 µg/L SeO_3^{2-}

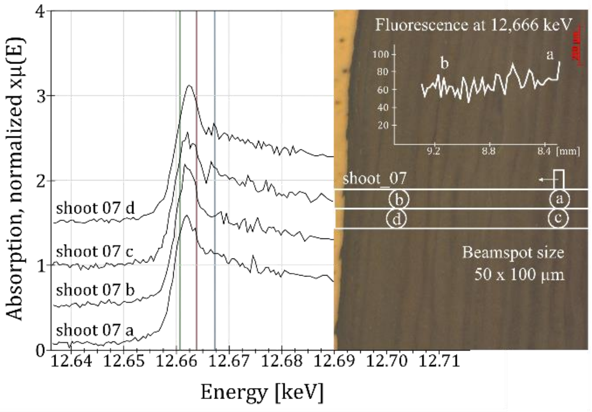

2000 µg/L SeO_3^{2-}

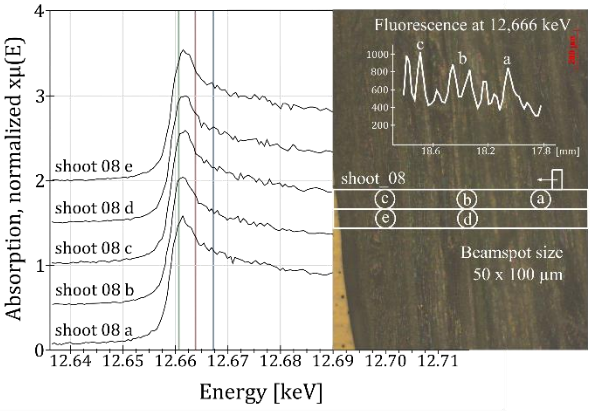

2000 µg/L SeO_3^{2-}

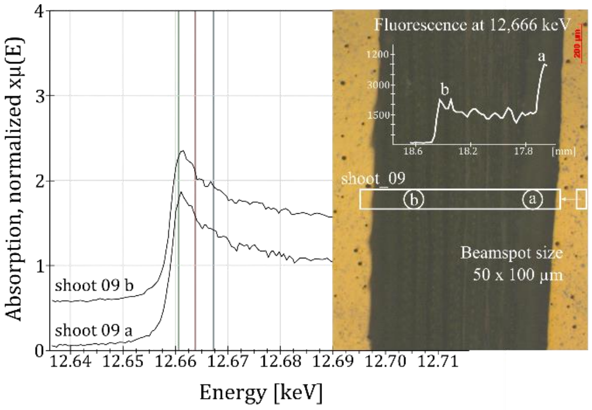

2000 µg/L SeO_3^{2-}

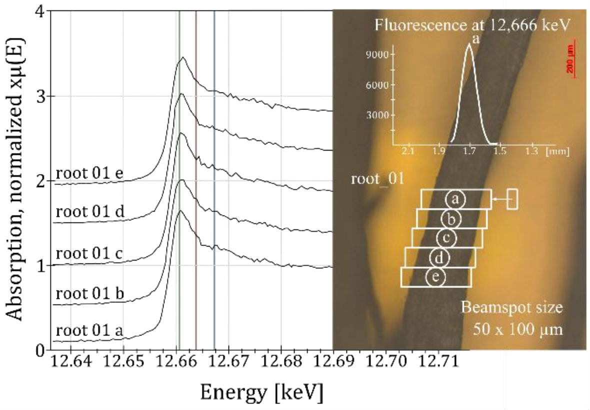

2000 µg/L SeO_3^{2-}

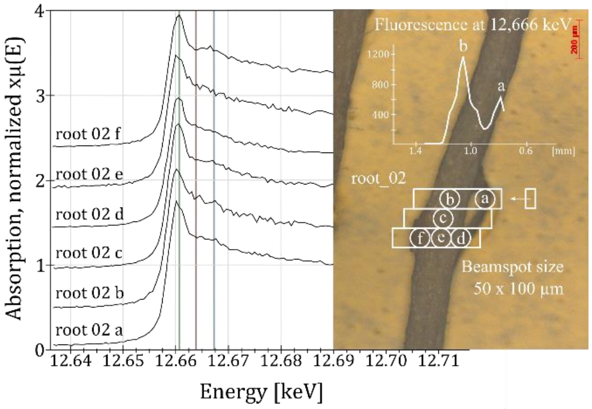

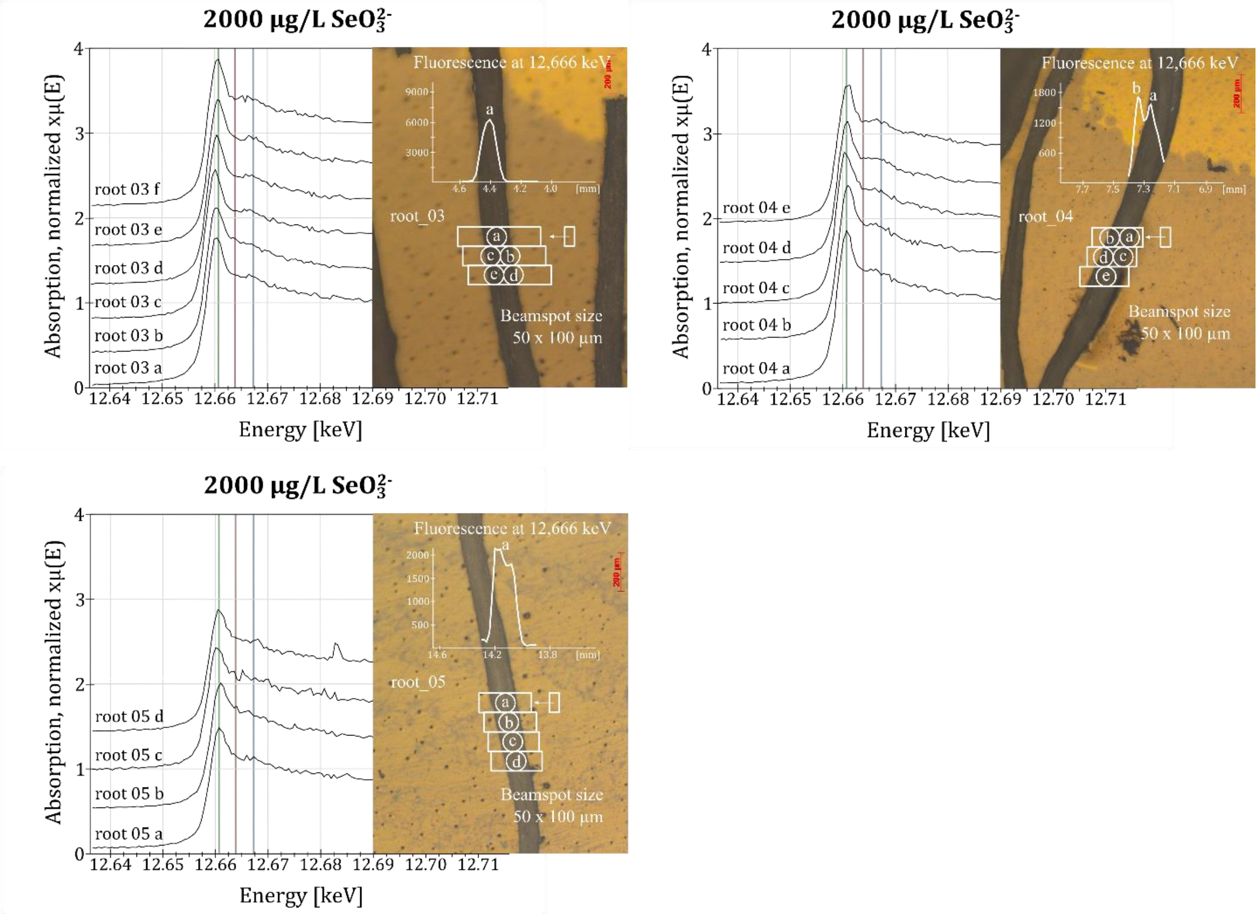

S21 Fig: all XANES spectra of each region of interest (ROI) on a rice plant treated with 2000 µg/L Se as Na_2SeO_3 in nutrient solution
