# Supplementary material for: Tracking Se Assimilation and Speciation through the Rice Plant – Nutrient Competition, Toxicity and Distribution
Source: PLoS One. 2016 Apr 26;11(4):e0152081. doi: 10.1371/journal.pone.0152081 (PMC4846085; doi:10.1371/journal.pone.0152081)
Supplement: S22 Fig — (PDF) [file pone.0152081.s022.pdf]

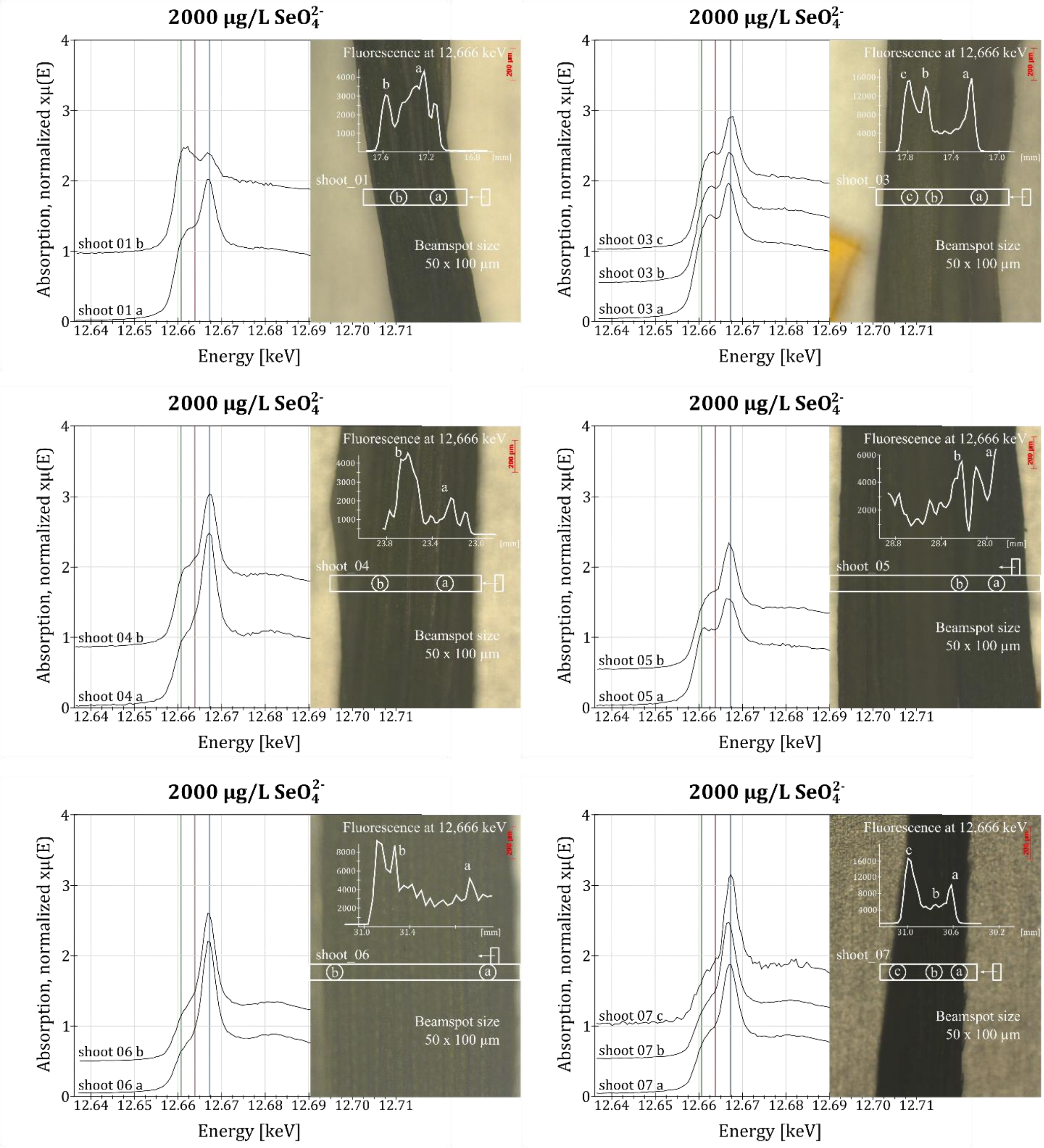
2000 µg/L $SeO_4^{2-}$
Absorption, normalized xµ(E)
Energy [keV]
Fluorescence at 12,666 keV
Beamspot size
50 x 100 µm
shoot 01 a
shoot 01 b
shoot_01
shoot 03 a
shoot 03 b
shoot 03 c
shoot_03
shoot 04 a
shoot 04 b
shoot_04
shoot 05 a
shoot 05 b
shoot_05
shoot 06 a
shoot 06 b
shoot_06
shoot 07 a
shoot 07 b
shoot 07 c
shoot_07
12.64 12.65 12.66 12.67 12.68 12.69 12.70 12.71
[mm]

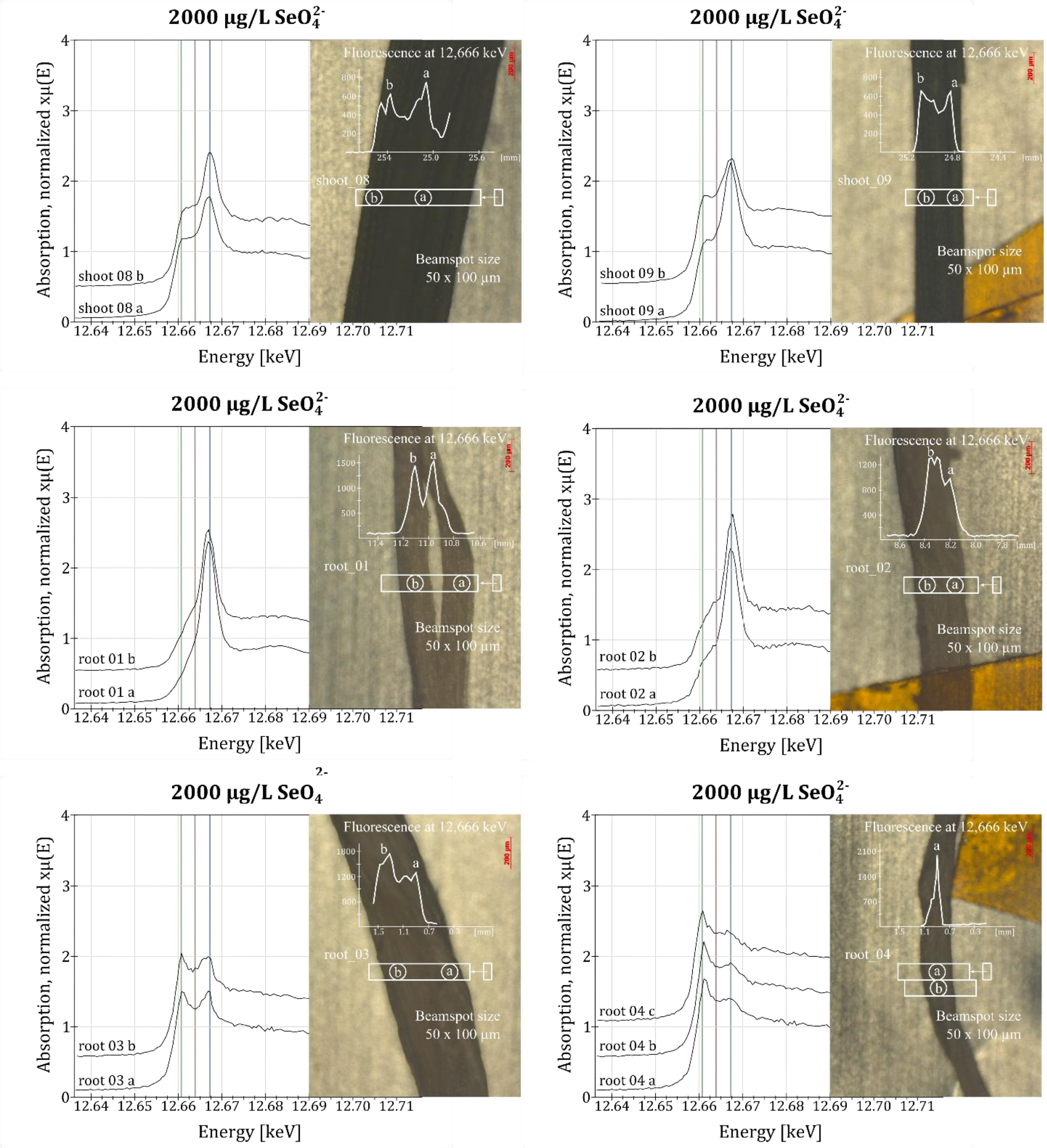


**S22 Fig: all XANES spectra of each region of interest (ROI) on a rice plant treated with 2000 µg/L Se as $Na_2SeO_4$ in nutrient solution**
