# Supplementary material for: Tracking Se Assimilation and Speciation through the Rice Plant – Nutrient Competition, Toxicity and Distribution
Source: PLoS One. 2016 Apr 26;11(4):e0152081. doi: 10.1371/journal.pone.0152081 (PMC4846085; doi:10.1371/journal.pone.0152081)
Supplement: S23 Fig — (PDF) [file pone.0152081.s023.pdf]

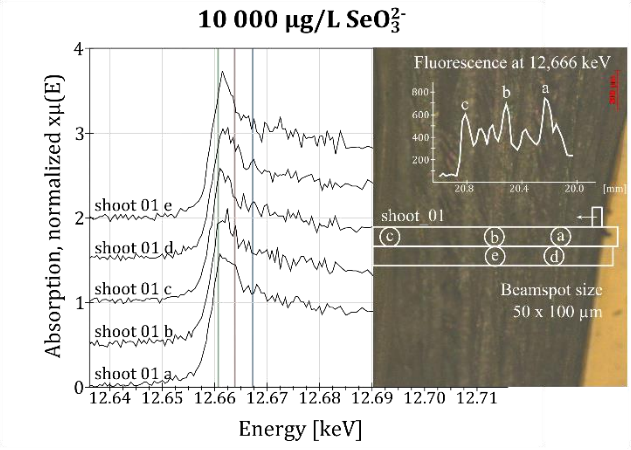
10 000 µg/L SeO_3^{2-}
Absorption, normalized xµ(E)
Energy [keV]
shoot 01 e
shoot 01 d
shoot 01 c
shoot 01 b
shoot 01 a
Fluorescence at 12,666 keV
shoot_01
Beamspot size
50 x 100 µm

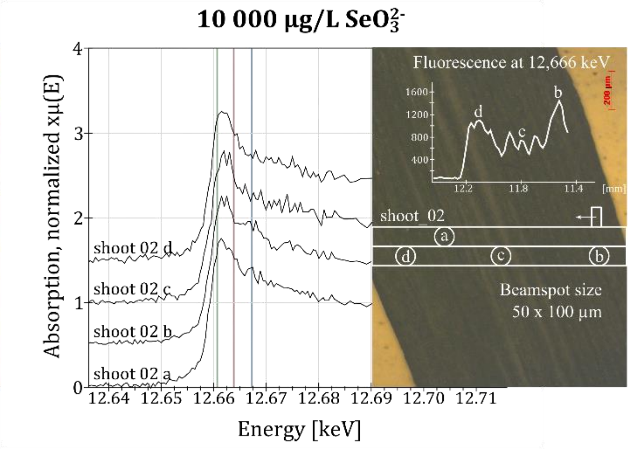
10 000 µg/L SeO_3^{2-}
Absorption, normalized xµ(E)
Energy [keV]
shoot 02 d
shoot 02 c
shoot 02 b
shoot 02 a
Fluorescence at 12,666 keV
shoot_02
Beamspot size
50 x 100 µm

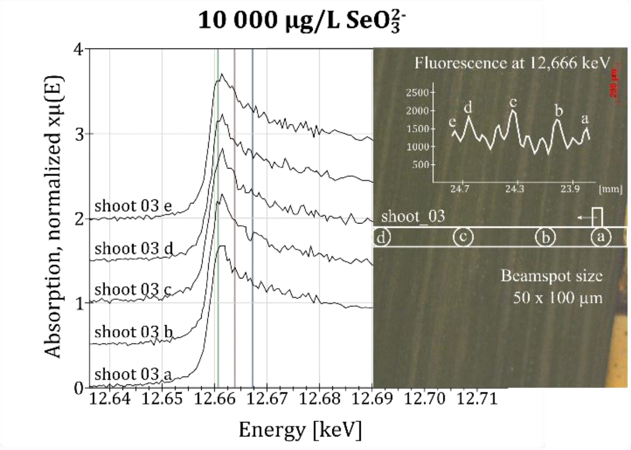
10 000 µg/L SeO_3^{2-}
Absorption, normalized xµ(E)
Energy [keV]
shoot 03 e
shoot 03 d
shoot 03 c
shoot 03 b
shoot 03 a
Fluorescence at 12,666 keV
shoot_03
Beamspot size
50 x 100 µm

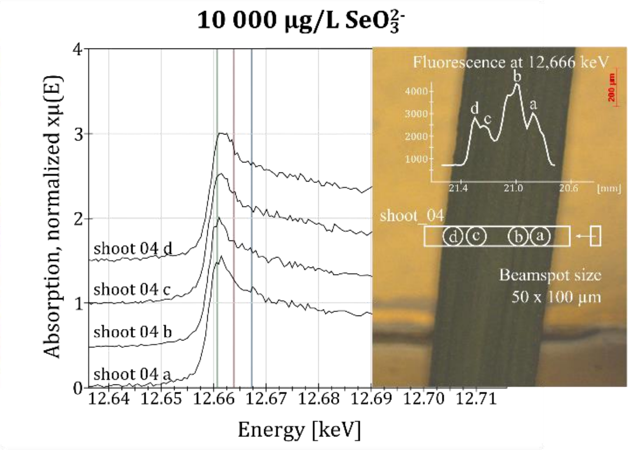
10 000 µg/L SeO_3^{2-}
Absorption, normalized xµ(E)
Energy [keV]
shoot 04 d
shoot 04 c
shoot 04 b
shoot 04 a
Fluorescence at 12,666 keV
shoot_04
Beamspot size
50 x 100 µm

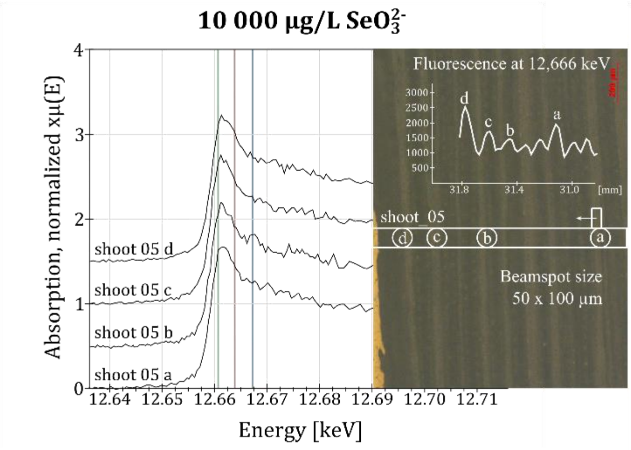
10 000 µg/L SeO_3^{2-}
Absorption, normalized xµ(E)
Energy [keV]
shoot 05 d
shoot 05 c
shoot 05 b
shoot 05 a
Fluorescence at 12,666 keV
shoot_05
Beamspot size
50 x 100 µm

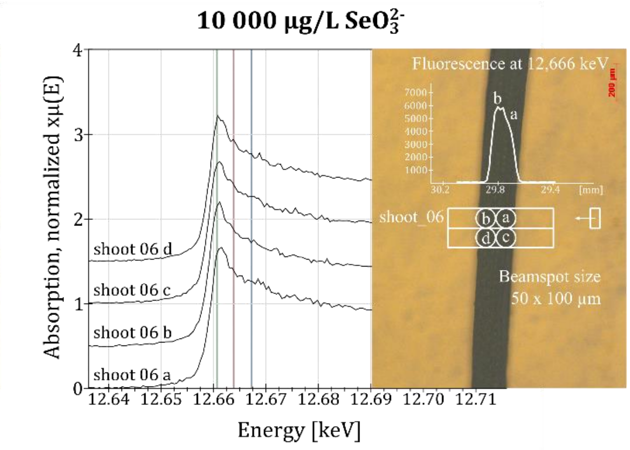
10 000 µg/L SeO_3^{2-}
Absorption, normalized xµ(E)
Energy [keV]
shoot 06 d
shoot 06 c
shoot 06 b
shoot 06 a
Fluorescence at 12,666 keV
shoot_06
Beamspot size
50 x 100 µm

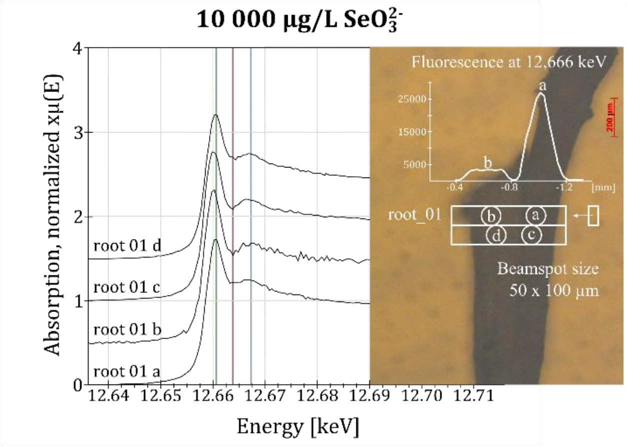
10 000 µg/L SeO_3^{2-}
Absorption, normalized xµ(E)
Energy [keV]
12.64 12.65 12.66 12.67 12.68 12.69 12.70 12.71
root 01 d
root 01 c
root 01 b
root 01 a
Fluorescence at 12,666 keV
root_01
Beamspot size
50 x 100 µm

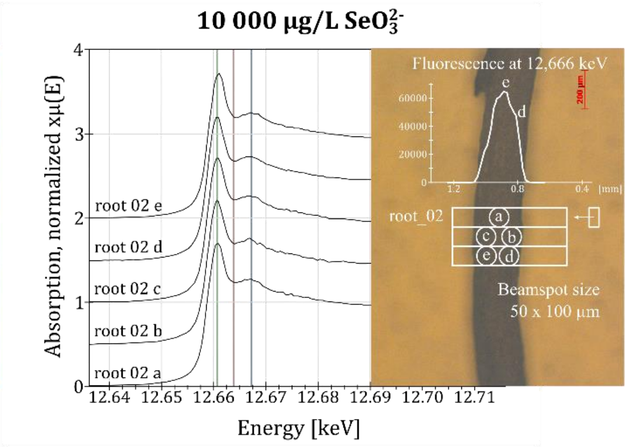
10 000 µg/L SeO_3^{2-}
Absorption, normalized xµ(E)
Energy [keV]
12.64 12.65 12.66 12.67 12.68 12.69 12.70 12.71
root 02 e
root 02 d
root 02 c
root 02 b
root 02 a
Fluorescence at 12,666 keV
root_02
Beamspot size
50 x 100 µm

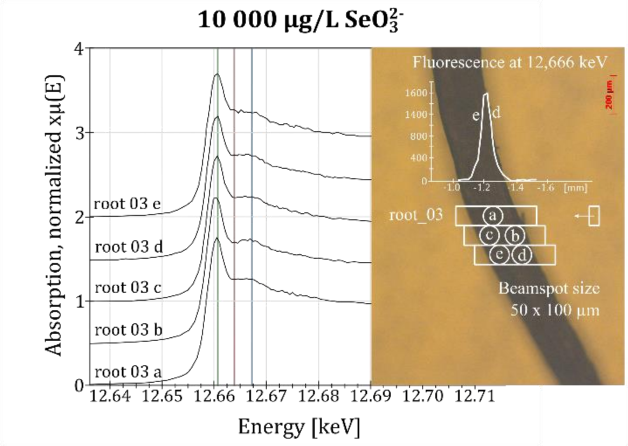
10 000 µg/L SeO_3^{2-}
Absorption, normalized xµ(E)
Energy [keV]
12.64 12.65 12.66 12.67 12.68 12.69 12.70 12.71
root 03 e
root 03 d
root 03 c
root 03 b
root 03 a
Fluorescence at 12,666 keV
root_03
Beamspot size
50 x 100 µm

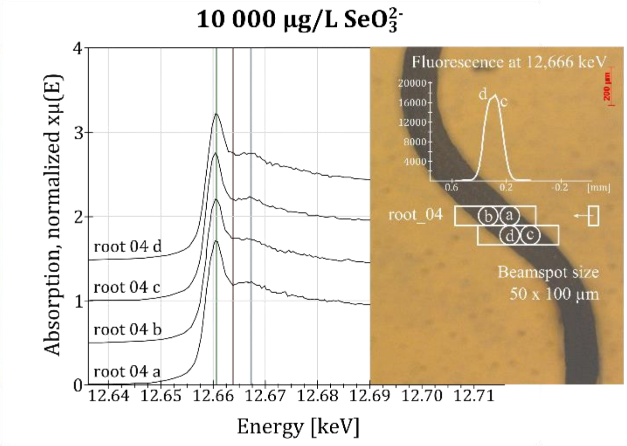
10 000 µg/L SeO_3^{2-}
Absorption, normalized xµ(E)
Energy [keV]
12.64 12.65 12.66 12.67 12.68 12.69 12.70 12.71
root 04 d
root 04 c
root 04 b
root 04 a
Fluorescence at 12,666 keV
root_04
Beamspot size
50 x 100 µm

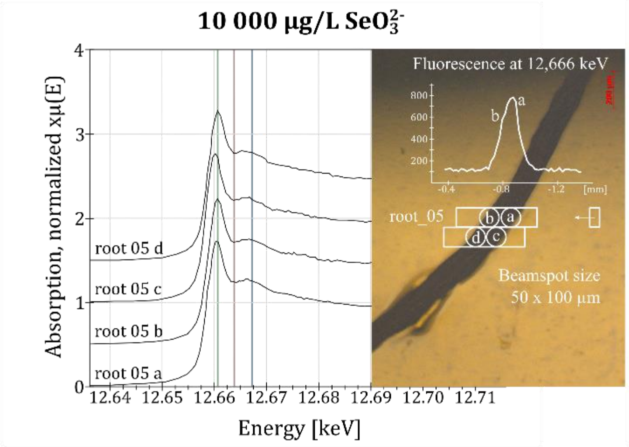
10 000 µg/L SeO_3^{2-}
Absorption, normalized xµ(E)
Energy [keV]
12.64 12.65 12.66 12.67 12.68 12.69 12.70 12.71
root 05 d
root 05 c
root 05 b
root 05 a
Fluorescence at 12,666 keV
root_05
Beamspot size
50 x 100 µm

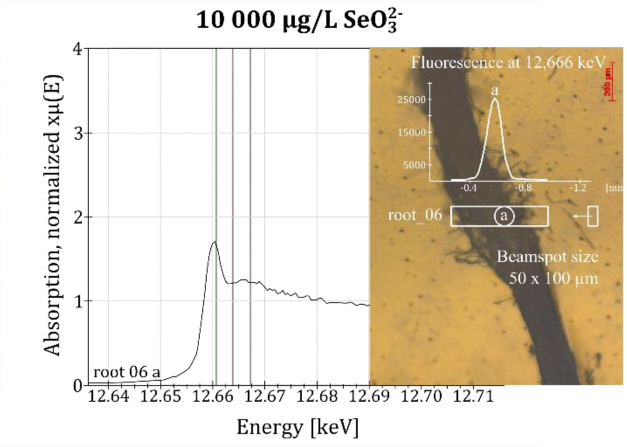
10 000 µg/L SeO_3^{2-}
Absorption, normalized xµ(E)
Energy [keV]
12.64 12.65 12.66 12.67 12.68 12.69 12.70 12.71
root 06 a
Fluorescence at 12,666 keV
root_06
Beamspot size
50 x 100 µm

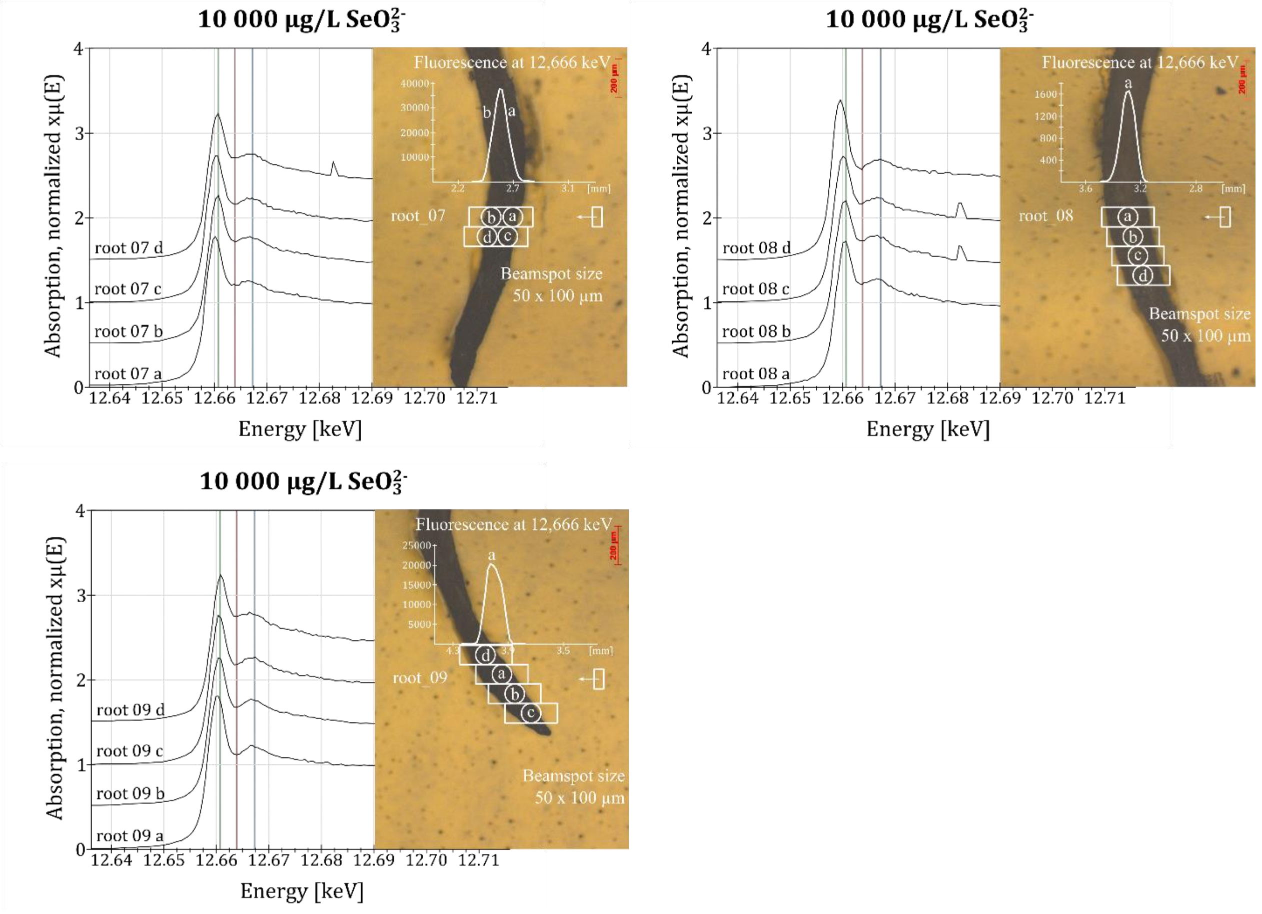

S23 Fig: all XANES spectra of each region of interest (ROI) on a rice plant treated with 10,000 µg/L Se as Na_2SeO_3 in nutrient solution
