# Supplementary material for: Tracking Se Assimilation and Speciation through the Rice Plant – Nutrient Competition, Toxicity and Distribution
Source: PLoS One. 2016 Apr 26;11(4):e0152081. doi: 10.1371/journal.pone.0152081 (PMC4846085; doi:10.1371/journal.pone.0152081)
Supplement: S1 Table — (PDF) [file pone.0152081.s025.pdf]

S1 Table: One-way ANOVA results for shoot-Se in agar plants when added as selenite

Groups (k)	Number (n)	Sum	Mean	Variance		
added c(Se) 0 µg/L	3	0.05	0.02	0.00		
added c(Se) 5 µg/L	3	1.08	0.36	0.39		
added c(Se) 10 µg/L	3	0.65	0.22	0.06		
added c(Se) 25 µg/L	3	2.35	0.78	0.46		
added c(Se) 50 µg/L	3	7.50	2.50	0.53		
added c(Se) 100 µg/L	3	9.87	3.29	1.76		
added c(Se) 250 µg/L	3	52.12	17.37	89.55		
added c(Se) 500 µg/L	3	90.12	30.04	75.60		
added c(Se) 1000 µg/L	3	189.21	63.07	495.78		
added c(Se) 2500 µg/L	3	433.89	144.63	1622.09		
**Distribution**	**Sum of squares (SS)**	**Degrees of freedom (df)**	**Mean sum of squares (MS)**	**Testing variable (F)**	**P-value**	**Critical F-value**
Difference between groups	57717.26	9.00	6413.03	28.05	1.95E-09	2.39
Difference within groups	4572.44	20.00	228.62			
total	62289.70	29.00				
