# Supplementary material for: Tracking Se Assimilation and Speciation through the Rice Plant – Nutrient Competition, Toxicity and Distribution
Source: PLoS One. 2016 Apr 26;11(4):e0152081. doi: 10.1371/journal.pone.0152081 (PMC4846085; doi:10.1371/journal.pone.0152081)
Supplement: S2 Table — (PDF) [file pone.0152081.s026.pdf]

S1 Table: One-way ANOVA results for root-Se in agar plants when added as selenite

Groups (k)	Number (n)	Sum	Mean	Variance		
added c(Se) 0 µg/L	3	0.00	0.00	0.00		
added c(Se) 5 µg/L	3	8.59	2.86	3.20		
added c(Se) 10 µg/L	3	17.80	5.93	7.88		
added c(Se) 25 µg/L	3	34.10	11.37	0.54		
added c(Se) 50 µg/L	3	78.80	26.27	314.83		
added c(Se) 100 µg/L	3	107.68	35.89	46.73		
added c(Se) 250 µg/L	3	189.47	63.16	785.79		
added c(Se) 500 µg/L	3	276.60	92.20	2370.37		
added c(Se) 1000 µg/L	3	531.93	177.31	4148.75		
added c(Se) 2500 µg/L	3	307.30	102.43	1964.41		
**Distribution**	**Sum of squares (SS)**	**Degrees of freedom (df)**	**Mean sum of squares (MS)**	**Testing variable (F)**	**P-value**	**Critical F-value**
Difference between groups	89398.66	9.00	9933.18	10.30	9.16E-06	2.39
Difference within groups	19285.03	20.00	964.25			
total	108683.69	29.00				
