# Supplementary material for: Tracking Se Assimilation and Speciation through the Rice Plant – Nutrient Competition, Toxicity and Distribution
Source: PLoS One. 2016 Apr 26;11(4):e0152081. doi: 10.1371/journal.pone.0152081 (PMC4846085; doi:10.1371/journal.pone.0152081)
Supplement: S3 Table — (PDF) [file pone.0152081.s027.pdf]

S1 Table: One-way ANOVA results for shoot-Se in agar plants when added as selenate

Groups (k)	Number (n)	Sum	Mean	Variance		
added c(Se) 0 µg/L	3	0.20	0.07	0.01		
added c(Se) 5 µg/L	3	18.54	6.18	5.08		
added c(Se) 10 µg/L	3	31.93	10.64	12.83		
added c(Se) 25 µg/L	3	112.00	37.33	503.22		
added c(Se) 50 µg/L	3	295.82	98.61	1564.10		
added c(Se) 100 µg/L	3	580.73	193.58	1589.16		
added c(Se) 250 µg/L	3	1099.92	366.64	7082.42		
added c(Se) 500 µg/L	3	429.53	143.18	3756.80		
added c(Se) 1000 µg/L	3	221.74	73.91	878.42		
added c(Se) 2500 µg/L	3	201.97	67.32	128.32		
**Distribution**	**Sum of squares (SS)**	**Degrees of freedom (df)**	**Mean sum of squares (MS)**	**Testing variable (F)**	**P-value**	**Critical F-value**
Difference between groups	342501.01	9.00	38055.67	24.52	6.53E-09	2.39
Difference within groups	31040.74	20.00	1552.04			
total	373541.76	29.00				
