# Supplementary material for: Tracking Se Assimilation and Speciation through the Rice Plant – Nutrient Competition, Toxicity and Distribution
Source: PLoS One. 2016 Apr 26;11(4):e0152081. doi: 10.1371/journal.pone.0152081 (PMC4846085; doi:10.1371/journal.pone.0152081)
Supplement: S4 Table — (PDF) [file pone.0152081.s028.pdf]

S1 Table: One-way ANOVA results for root-Se in agar plants when added as selenate

Groups (k)	Number (n)	Sum	Mean	Variance		
added c(Se) 0 µg/L	3	0.09	0.03	0.00		
added c(Se) 5 µg/L	3	5.49	1.83	0.13		
added c(Se) 10 µg/L	3	9.95	3.32	0.07		
added c(Se) 25 µg/L	3	26.76	8.92	26.09		
added c(Se) 50 µg/L	3	64.74	21.58	4.13		
added c(Se) 100 µg/L	3	134.86	44.95	205.35		
added c(Se) 250 µg/L	3	286.65	95.55	1500.89		
added c(Se) 500 µg/L	3	287.26	95.75	2250.82		
added c(Se) 1000 µg/L	3	232.32	77.44	913.02		
added c(Se) 2500 µg/L	3	201.53	67.18	1047.96		
**Distribution**	**Sum of squares (SS)**	**Degrees of freedom (df)**	**Mean sum of squares (MS)**	**Testing variable (F)**	**P-value**	**Critical F-value**
Difference between groups	42112.64	9.00	4679.18	7.87	6.75E-05	2.39
Difference within groups	11896.98	20.00	594.85			
total	54009.62	29.00				
