# Supplementary material for: Tracking Se Assimilation and Speciation through the Rice Plant – Nutrient Competition, Toxicity and Distribution
Source: PLoS One. 2016 Apr 26;11(4):e0152081. doi: 10.1371/journal.pone.0152081 (PMC4846085; doi:10.1371/journal.pone.0152081)
Supplement: S5 Table — (PDF) [file pone.0152081.s029.pdf]

S1 Table: One-way ANOVA results for shoot-Se in nut.sol. plants when added as selenite

Groups (k)	Number (n)	Sum	Mean	Variance		
added c(Se) 0 µg/L	3	0.00	0.00	0.00		
added c(Se) 5 µg/L	3	1.24	0.41	0.03		
added c(Se) 10 µg/L	3	2.90	0.97	0.18		
added c(Se) 25 µg/L	3	7.05	2.35	0.34		
added c(Se) 50 µg/L	3	16.73	5.58	6.06		
added c(Se) 100 µg/L	3	28.74	9.58	3.19		
added c(Se) 250 µg/L	3	85.25	28.42	17.20		
added c(Se) 500 µg/L	3	178.01	59.34	80.16		
added c(Se) 1000 µg/L	3	305.72	101.91	833.96		
added c(Se) 2500 µg/L	3	476.95	158.98	922.72		
**Distribution**	**Sum of squares (SS)**	**Degrees of freedom (df)**	**Mean sum of squares (MS)**	**Testing variable (F)**	**P-value**	**Critical F-value**
Difference between groups	79832.70	9.00	8870.30	47.59	1.48E-11	2.39
Difference within groups	3727.68	20.00	186.38			
total	83560.38	29.00				
