# Supplementary material for: Tracking Se Assimilation and Speciation through the Rice Plant – Nutrient Competition, Toxicity and Distribution
Source: PLoS One. 2016 Apr 26;11(4):e0152081. doi: 10.1371/journal.pone.0152081 (PMC4846085; doi:10.1371/journal.pone.0152081)
Supplement: S6 Table — (PDF) [file pone.0152081.s030.pdf]

S1 Table: One-way ANOVA results for root-Se in nut.sol. plants when added as selenite

Groups (k)	Number (n)	Sum	Mean	Variance		
added c(Se) 0 µg/L	3	0.00	0.00	0.00		
added c(Se) 5 µg/L	3	3.21	1.07	0.29		
added c(Se) 10 µg/L	3	5.80	1.93	0.86		
added c(Se) 25 µg/L	3	16.54	5.51	7.02		
added c(Se) 50 µg/L	3	66.05	22.02	184.57		
added c(Se) 100 µg/L	3	80.12	26.71	230.07		
added c(Se) 250 µg/L	3	168.37	56.12	31.06		
added c(Se) 500 µg/L	3	420.40	140.13	1259.38		
added c(Se) 1000 µg/L	3	534.42	178.14	1429.71		
added c(Se) 2500 µg/L	3	936.13	312.04	214.73		
**Distribution**	**Sum of squares (SS)**	**Degrees of freedom (df)**	**Mean sum of squares (MS)**	**Testing variable (F)**	**P-value**	**Critical F-value**
Difference between groups	293460.48	9.00	32606.72	97.11	1.60E-14	2.39
Difference within groups	6715.38	20.00	335.77			
total	300175.86	29.00				
