# Supplementary material for: Tracking Se Assimilation and Speciation through the Rice Plant – Nutrient Competition, Toxicity and Distribution
Source: PLoS One. 2016 Apr 26;11(4):e0152081. doi: 10.1371/journal.pone.0152081 (PMC4846085; doi:10.1371/journal.pone.0152081)
Supplement: S7 Table — (PDF) [file pone.0152081.s031.pdf]

S1 Table: One-way ANOVA results for shoot-Se in nut.sol. plants when added as selenate

Groups (k)	Number (n)	Sum	Mean	Variance		
added c(Se) 0 µg/L	3	0.19	0.06	0.01		
added c(Se) 5 µg/L	3	1.74	0.58	0.21		
added c(Se) 10 µg/L	3	2.26	0.75	0.03		
added c(Se) 25 µg/L	3	6.59	2.20	0.07		
added c(Se) 50 µg/L	3	17.26	5.75	2.06		
added c(Se) 100 µg/L	3	27.22	9.07	25.86		
added c(Se) 250 µg/L	3	121.74	40.58	114.77		
added c(Se) 500 µg/L	3	244.91	81.64	204.99		
added c(Se) 1000 µg/L	3	573.13	191.04	47.94		
added c(Se) 2500 µg/L	3	1216.02	405.34	3415.09		
**Distribution**	**Sum of squares (SS)**	**Degrees of freedom (df)**	**Mean sum of squares (MS)**	**Testing variable (F)**	**P-value**	**Critical F-value**
Difference between groups	464727.90	9.00	51636.43	135.49	6.22E-16	2.39
Difference within groups	7622.08	20.00	381.10			
total	472349.98	29.00				
