# Supplementary material for: Tracking Se Assimilation and Speciation through the Rice Plant – Nutrient Competition, Toxicity and Distribution
Source: PLoS One. 2016 Apr 26;11(4):e0152081. doi: 10.1371/journal.pone.0152081 (PMC4846085; doi:10.1371/journal.pone.0152081)
Supplement: S9 Table — (PDF) [file pone.0152081.s033.pdf]

S1 Table: Se distribution for uptake of selenate and selenite into root and shoot in all three experimental set-ups (d_{shoot} [%] = $c(Se)_{shoot}$ [mg/kg]/($c(Se)_{shoot}$ [mg/kg] + $c(Se)_{root}$ [mg/kg])*100)

	Nutrient-free, direct Se-uptake						Nutrient-free, Se-uptake delayed						Nutrient solution, Se-uptake delayed					
	Na_2SeO_4			Na_2SeO_3			Na_2SeO_4			Na_2SeO_3			Na_2SeO_4			Na_2SeO_3		
c(Se)	shoot	root	SD	shoot	root	SD	shoot	root	SD	shoot	root	SD	shoot	root	SD	shoot	root	SD
[µ/L]	[%]	[%]	± s	[%]	[%]	± s	[%]	[%]	± s	[%]	[%]	± s	[%]	[%]	± s	[%]	[%]	± s
5	38	62	7	77	23	6	28	72	8	62	38	28	81	19	3	26	74	3
10	32	68	7	76	24	8	33	67	1	78	22	19	78	22	3	27	73	5
25	37	63	7	81	19	11	30	70	8	74	26	21	83	17	2	29	71	2
50	32	68	10	82	18	7	20	80	11	70	30	1	78	22	2	31	69	3
100	38	62	16	81	19	9	26	74	12	73	27	7	79	21	2	26	74	5
250	38	62	8	79	21	11	34	66	3	70	30	10	79	21	2	23	77	5
500	34	66	3	60	40	5	30	70	3	73	27	9	79	21	3	23	77	4
1000	30	70	8	49	51	16	36	64	3	75	25	6	67	33	2	32	68	4
2500	35	65	6	50	50	16	34	66	3	76	24	8	53	47	4	20	80	3
