# Supplementary material for: Tracking Se Assimilation and Speciation through the Rice Plant – Nutrient Competition, Toxicity and Distribution
Source: PLoS One. 2016 Apr 26;11(4):e0152081. doi: 10.1371/journal.pone.0152081 (PMC4846085; doi:10.1371/journal.pone.0152081)
Supplement: S10 Table — (PDF) [file pone.0152081.s034.pdf]

S1 Table: Wet weight accumulation factors AF of selenate into plant tissue in all three experimental set-ups (AF [-] = $c(Se)_{medium}$ [mg/L] / $c(Se)_{plant}$ [mg/kg])

	Nutrient-free, direct Se-uptake				Nutrient-free, Se-uptake delayed				Nutrient solution, Se-uptake delayed			
c(Se)	shoot	SD	root	SD	shoot	SD	root	SD	shoot	SD	root	SD
[µ/L]	[-]	± s	[-]	± s	[-]	± s	[-]	± s	[-]	± s	[-]	± s
5	274	26	108	6	376	15	165	10	27	21	22	38
10	263	31	99	2	332	20	192	21	16	3	8	9
25	390	196	130	41	297	8	144	12	21	2	10	8
50	465	42	155	22	644	23	373	9	27	10	13	5
100	514	55	175	13	786	33	459	27	19	8	10	5
250	366	42	132	35	779	35	416	17	41	13	20	11
500	94	59	80	32	651	48	427	15	40	11	22	3
1000	25	6	37	11	478	26	434	15	53	19	26	14
2500	8	2	13	5	50	6	123	5	41	9	23	8
