# Supplementary material for: Tracking Se Assimilation and Speciation through the Rice Plant – Nutrient Competition, Toxicity and Distribution
Source: PLoS One. 2016 Apr 26;11(4):e0152081. doi: 10.1371/journal.pone.0152081 (PMC4846085; doi:10.1371/journal.pone.0152081)
Supplement: S11 Table — (PDF) [file pone.0152081.s035.pdf]

S11 Table: Wet weight accumulation factors AF of selenite into plant tissue in all three experimental set-ups (AF [-] = $c(Se)_{medium}$ [mg/L] / $c(Se)_{plant}$ [mg/kg])

	Nutrient-free, direct Se-uptake				Nutrient-free, Se-uptake delayed				Nutrient solution, Se-uptake delayed			
c(Se)	shoot	SD	root	SD	shoot	SD	root	SD	shoot	SD	root	SD
[µ/L]	[-]	± s	[-]	± s	[-]	± s	[-]	± s	[-]	± s	[-]	± s
5	75	30	198	147	116	14	428	2	22	10	80	50
10	53	17	135	29	77	10	267	25	27	12	82	43
25	59	20	130	16	105	10	262	2	27	7	99	32
50	50	18	145	62	111	6	357	27	32	14	212	99
100	53	25	147	71	90	7	345	39	27	6	105	48
250	33	12	32	29	81	13	246	23	29	2	94	34
500	28	18	76	44	39	6	138	6	34	13	117	97
1000	23	12	69	26	30	2	92	7	31	13	85	49
2500	7	2	18	7	10	1	40	2	22	8	58	14
