# Supplementary material for: Tracking Se Assimilation and Speciation through the Rice Plant – Nutrient Competition, Toxicity and Distribution
Source: PLoS One. 2016 Apr 26;11(4):e0152081. doi: 10.1371/journal.pone.0152081 (PMC4846085; doi:10.1371/journal.pone.0152081)
Supplement: S12 Table — (PDF) [file pone.0152081.s036.pdf]

S1 Table: Linear Combination Fitting Results using references for selenomethionine (org. Se), selenite and selenate

Se as Na_2SeO_3

**500 µg/L**	**org. Se [%]**	**selenite [%]**	**selenate [%]**	**R-factor [-]**
root 01	73	27	0	1.57
root 02	76	24	0	1.07
root 03	79	21	0	0.96
root 04	84	16	0	0.78
root 05	85	15	0	0.67
root 06	91	9	0	0.56
root 07	100	0	0	0.51
root 08	92	8	0	0.71
**mean**	85	15	0	0.85
**SD**	9	9	0	0.35
shoot 01	64	36	0	1.97
shoot 02	63	37	0	3.59
shoot 03	67	33	0	1.63
shoot 04	74	26	0	1.22
shoot 05	71	29	0	2.94
shoot 06	50	50	0	2.20
shoot 07	65	35	0	2.25
**mean**	65	35	0	2.26
**SD**	8	8	0	0.80
**2000 µg/L**	**org. Se [%]**	**selenite [%]**	**selenate [%]**	**R-factor [-]**
root 01	89	11	0	0.43
root 02	97	1	2	0.48
root 03	100	0	0	0.62
root 04	97	0	3	0.34
root 05	94	4	2	0.48
**mean**	95	3	1	0.47
**SD**	4	5	1	0.10
shoot 01	69	31	0	2.31
shoot 02	63	37	0	3.59
shoot 03	73	27	0	1.11
shoot 04	68	32	0	1.54
shoot 05	69	31	0	1.76
shoot 06	76	24	0	1.01
shoot 07	69	31	0	2.57
shoot 08	75	25	0	0.92
shoot 09	100	0	0	1.78
**mean**	73	27	0	1.84
**SD**	11	11	0	0.86
**10000 µg/L**	**org. Se [%]**	**selenite [%]**	**selenate [%]**	**R-factor [-]**
root 01	100	0	0	1.38
root 02	95	3	3	0.84
root 03	100	0	0	0.74
root 04	100	0	0	0.89
root 05	99	1	0	0.97
root 06	100	0	0	1.87
root 07	100	0	0	1.26
root 08	100	0	0	1.98
root 09	100	0	0	1.35
**mean**	99	0	0	1.25
**SD**	2	1	1	0.44
shoot 01	75	25	0	1.12
shoot 02	74	26	0	0.97
shoot 03	81	19	0	0.62
shoot 04	81	19	0	0.57
shoot 05	76	24	0	0.87
shoot 06	81	19	0	0.70
**mean**	78	22	0	0.81
**SD**	3	3	0	0.21

Se as Na_2SeO_4

**500 µg/L**	**org. Se [%]**	**selenite [%]**	**selenate [%]**	**R-factor [-]**
root 01	31	6	63	0.75
root 02	32	6	62	0.89
root 04	53	17	30	1.08
root 06	50	18	32	1.23
root 07	43	16	41	0.47
**mean**	42	13	46	0.88
**SD**	10	6	16	0.29
shoot 01	45	23	32	2.02
shoot 02	33	9	58	1.25
shoot 03	41	28	31	1.39
shoot 04	30	6	65	0.38
shoot 05	56	11	32	0.46
shoot 06	27	6	67	0.65
shoot 07	33	16	51	0.88
**mean**	38	14	48	1.00
**SD**	10	9	16	0.59
**2000 µg/L**	**org. Se [%]**	**selenite [%]**	**selenate [%]**	**R-factor [-]**
root 01	14	15	71	0.92
root 02	32	10	57	0.55
root 03	64	27	9	0.71
root 05	80	15	5	0.55
**mean**	48	17	36	0.68
**SD**	30	7	33	0.17
shoot 01	56	22	22	0.54
shoot 03	56	22	22	0.54
shoot 04	38	18	44	0.54
shoot 05	50	20	30	0.36
shoot 06	25	10	65	1.45
shoot 07	33	17	50	0.65
shoot 08	52	18	30	0.52
shoot 09	48	13	39	0.42
**mean**	45	18	38	0.63
**SD**	11	4	15	0.34
**10000 µg/L**	**org. Se [%]**	**selenite [%]**	**selenate [%]**	**R-factor [-]**
root 01	43	6	51	0.39
root 02	47	8	44	0.32
root 03	57	31	12	0.89
root 04	68	23	8	0.66
**mean**	54	17	29	0.56
**SD**	11	12	22	0.26
shoot 01	58	20	22	0.52
shoot 02	56	13	32	0.52
shoot 03	66	22	11	0.51
shoot 04	52	12	36	0.45
shoot 05	48	10	43	0.41
shoot 06	50	7	43	0.40
shoot 07	50	9	41	0.29
**mean**	54	13	32	0.44
**SD**	7	6	12	0.09
